# Transcriptomic analyses of bacterial growth on fungal necromass reveal different microbial community niches during degradation

**DOI:** 10.1128/aem.01062-24

**Published:** 2024-09-12

**Authors:** Jessica K. Novak, Peter G. Kennedy, Jeffrey G. Gardner

**Affiliations:** 1 Department of Biological Sciences, University of Maryland—Baltimore County, Baltimore, Maryland, USA; 2 Department of Plant and Microbial Biology, University of Minnesota, Minneapolis, Minnesota, USA; Danmarks Tekniske Universitet The Novo Nordisk Foundation Center for Biosustainability, Kgs. Lyngby, Denmark

**Keywords:** carbohydrate active enzyme, *Cellvibrio japonicus*, *Chitinophaga pinensis*, *Hyaloscypha bicolor*, necromass, *Serratia marcescens*

## Abstract

Bacteria are major drivers of organic matter decomposition and play crucial roles in global nutrient cycling. Although the degradation of dead fungal biomass (necromass) is increasingly recognized as an important contributor to soil carbon (C) and nitrogen (N) cycling, the genes and metabolic pathways involved in necromass degradation are less characterized. In particular, how bacteria degrade necromass containing different quantities of melanin, which largely control rates of necromass decomposition *in situ*, is largely unknown. To address this gap, we conducted a multi-timepoint transcriptomic analysis using three Gram-negative, bacterial species grown on low or high melanin necromass of *Hyaloscypha bicolor*. The bacterial species, *Cellvibrio japonicus, Chitinophaga pinensis*, and *Serratia marcescens*, belong to genera known to degrade necromass *in situ*. We found that while bacterial growth was consistently higher on low than high melanin necromass, the CAZyme-encoding gene expression response of the three species was similar between the two necromass types. Interestingly, this trend was not shared for genes encoding nitrogen utilization, which varied in *C. pinensis* and *S. marcescens* during growth on high vs low melanin necromass. Additionally, this study tested the metabolic capabilities of these bacterial species to grow on a diversity of C and N sources and found that the three bacteria have substantially different utilization patterns. Collectively, our data suggest that as necromass changes chemically over the course of degradation, certain bacterial species are favored based on their differential metabolic capacities.

**IMPORTANCE:**

Fungal necromass is a major component of the carbon (C) in soils as well as an important source of nitrogen (N) for plant and microbial growth. Bacteria associated with necromass represent a distinct subset of the soil microbiome and characterizing their functional capacities is the critical next step toward understanding how they influence necromass turnover. This is particularly important for necromass varying in melanin content, which has been observed to control the rate of necromass decomposition across a variety of ecosystems. Here we assessed the gene expression of three necromass-degrading bacteria grown on low or high melanin necromass and characterized their metabolic capacities to grow on different C and N substrates. These transcriptomic and metabolic studies provide the first steps toward assessing the physiological relevance of up-regulated CAZyme-encoding genes in necromass decomposition and provide foundational data for generating a predictive model of the molecular mechanisms underpinning necromass decomposition by soil bacteria.

## INTRODUCTION

Ectomycorrhizal (ECM) fungi are major drivers of soil nutrient cycling in forest ecosystems worldwide (1–3). Through their extensive mycelial networks and diverse enzymatic capacities, ECM fungi scavenge nutrients from sources and spaces in soil that are largely inaccessible to plants (4, 5). These nutrients are then traded with plant hosts in exchange for photosynthetically derived sugars (6). This interaction ends with the death of either the plant or the ECM fungus, and the resulting organic material enters the brown food web as a substrate for decomposition. While carbon utilization and nutrient recovery from dead plant material by soil microbes are well characterized, much less known about microbial resource acquisition derived from dead ECM fungi (7, 8) despite previous knowledge that fungal necromass decomposition can be a significant contributor to soil carbon and nutrient cycling (9, 10).

It is hypothesized that fungal cell wall components are the major sources of carbon and nutrition available in necromass (11). The carbohydrate component of necromass includes β-glucans, α-chitin, and α-mannans, and these substrates possess β-1,3- and β-1,6-linked glucose, β-1,4-linked *N*-acetylglucosamine, and α-1,2-linked mannose, respectively (12). In terms of fungal cell wall structure, the outermost layer is often the thickest and largely comprised of α-mannans and β-glucans, with α-chitin deeper within the cell wall and closer to the membrane (13). Necromass also frequently contains melanin (14) which is a complex polymer derived from 3,4-dihydroxyphenylalanine (DOPA) that is intermeshed among the polysaccharides of the fungal cell wall (15–17). Notably, previous studies on fungal cell wall degradation found that a high melanin content slowed the decomposition rate because it limited access to polysaccharides and/or disrupted the membrane of the microbial decomposers (18, 19).

In this study, we used the necromass of *Hyaloscypha bicolor,* formerly known as *Meliniomyces bicolor* (20). The rationale of using this fungus is that it can be naturally manipulated to have either low or high melanin phenotypes (21), which have been consistently demonstrated to have different rates of decay (22, 23). Previous work on *H. bicolor* degradation by bacterial decomposers found changes in the necrobiome community based on melanin content, stage of decay, and the associated plant host (21). The dominating bacterial decomposers included genera such as *Chitinophaga, Pseudomonas, Cellvibrio, Burkholderia,* and *Luteibacter* (21). However, identifying the contribution of individual bacterial genera to fungal cell wall degradation has been understudied (24). Consequently, there is a need to investigate the saccharifying capabilities of necromass-degrading bacteria to assemble a working model of *H. bicolor* decomposition that includes in both spatial and temporal aspects.

To begin our study, we measured the growth and gene expression of three bacterial species using both high and low melanin phenotypes of *H. bicolor* necromass. The bacterial species (*Cellvibrio japonicus* Ueda107*, Chitinophaga pinensis* DSM2588*,* and *Serratia marcescens* PIC3611) were selected because they are proficient polysaccharide degraders, and members of these three genera have been previously identified as being associated with decaying necromass *in situ* (21). Another shared characteristic of these genera is their expansive suite of carbohydrate-active enzymes (CAZymes), which are able to hydrolyze the diverse glycosidic linkages found in fungal cell wall polysaccharides (25–28). Examples of CAZymes required for fungal cell wall degradation include glycoside hydrolases (GHs), lytic polysaccharides monooxygenases (LPMOs), and carbohydrate esterases (CEs) (29–31).

By assessing the gene expression of each bacterial species grown on *H. bicolor* necromass, we identified CAZyme-encoding gene targets with likely physiological importance for necromass decomposition. Additionally, comparing the growth dynamics and expression responses of the three bacterial species on high vs low melanin *H. bicolor* necromass helped explain the decelerated decay rate of high melanin necromass. In terms of growth capabilities, we found that the bacterial growth rates were generally slower on high melanin necromass and often resulted in a lower final cell density. However, the CAZyme-encoding gene expression responses were almost identical for all three species on both the high and low melanin necromass. These gene expression analyses also revealed that *C. pinensis* gene expression was regulated both temporally and by substrate, whereas *C. japonicus* and *S. marcescens* CAZyme gene expression was largely regulated by substrate. Furthermore, we performed a phenotypic microarray to identify substrate utilization for each bacterial species on 190 carbon sources and 95 nitrogen sources and found that *S. marcescens* is metabolically active on a much broader array of sole nitrogen sources compared to the other two strains. Overall, our results identified distinct bioconversion differences among the bacterial species, which aided in the generation of a model for necromass degradation by bacteria.

## MATERIALS AND METHODS

### Necromass preparation

*Hyaloscypha bicolor* (formerly *Meliniomyces bicolor*) cultures were grown on half-strength potato dextrose (PD; HiMedia Laboratories, PA, USA) agar plates covered with gel drying film (Promega, WI, USA). *H. bicolor* cultures were maintained at 23°C in the dark for 3 weeks. Mycelial plugs were transferred to liquid PD broth with pH adjusted to 5 using 10% HCl. Cultures were grown in 125 mL flasks filled with either 40 or 110 mL for low and high melanin biomass, respectively. The cultures were then grown in shaking incubators (120 RPM for low melanin and 150 RPM for high melanin) for 30 days at 25°C. *H. bicolor* mycelium were then harvested in bulk onto sterile sieves and rinsed with sterile deionized water. Next, the mycelium of each melanization level was homogenized using a sterilized mortar and pestle, transferred to sterile 50 mL centrifuge tubes (Fisherbrand, PA, USA), and stored at −80°C overnight. Tubes were then placed into a benchtop Freeze Dryer (Labconco, NH, USA) for 3 days at −50°C under vacuum to create the necromass used in this study.

### Bacterial growth conditions

*C. japonicus* Ueda107*, C. pinensis* DSM2588*,* and *S. marcescens* PIC3611 strains were grown in MOPS-defined media (TekNova #M2106), supplemented with 1.3 mM phosphate and 0.2% (wt/vol) glucose per manufacturer’s instructions. Strains propagated in liquid culture were grown at 30°C with high aeration (200 RPM) as done previously (32). Plate media was solidified with 1.5% (wt/vol) agar. Strains used for growth analyses were grown overnight in 5 mL MOPS-glucose broth until full density was reached (OD_600_ ~1.5), then diluted 1:100 into MOPS-defined media containing 1.3 mM phosphate and either high or low melanin necromass at a concentration of 1% (wt/vol). The insoluble necromass was contained in 90 µm nylon mesh bags (The Press Club #B079S6JNQW). The necromass was autoclaved in deionized water (30 min steam cycle; 121°C, 16 psi) and then rinsed twice with sterile deionized water before use in growth experiments.

### Transcriptome sampling and analysis

Transcriptome sampling was conducted as previously described (32). Briefly, cultures were grown in 500 mL flasks in biological triplicate with MOPS-defined media and either 0.2% (wt/vol) glucose or 1% (wt/vol) necromass. Optical density (OD_600_) measurements were taken to capture the growth dynamics of the three bacterial species on each substrate over 4 days. Sampling occurred during the mid-exponential growth and stationary phase, where cultures that reached mid-exponential growth at OD_600_ ≥ 0.1, then 35 mL of culture was aseptically collected and added to 5 mL phenol:ethanol (5:95; vol/vol) to stop metabolism. In cultures with mid-exponential growth OD_600_ ≤ 0.1, 70 mL of culture as added to a 10 mL phenol:ethanol (5:95 vol/vol) to ensure sufficient cell mass was obtained. The metabolically stopped cell suspensions were then immediately centrifuged at 8,000 × *g* for 5 min at 4°C. Supernatants were removed, and cell pellet was flash-frozen in a dry ice and ethanol bath for 5 min before storage at −80°C. Frozen cell pellets were sent to Azenta (South Plainfield, NJ) for mRNA extraction, mRNA QA, cDNA library preparation, and RNAseq. All samples had an RIN above 8.0 during QA. Sequencing used an Illumina HiSeq and generated ~350 M pair-end (2 × 150 bp), single index reads, and ~90% of bases > Q30 with a mean quality score >35. The raw FASTQ files were quality checked, aligned to existing genomes, and analyzed for differential expression using the Galaxy platform (33). Unless otherwise noted below, default parameters were in all analysis tools used except in cases where strandedness was specified (all RNAseq data were unstranded). Briefly, RNAseq data were concatenated using the concatenate data sets: tail-to-head (cat) tool (34). Transcript quality was tested using FastQC, which indicated read trimming was unnecessary for any of the files (35). Transcripts were next aligned to their respective reference genomes using the HISAT2 tool (36) and quantified using the htseq-count tool (37). Reference genome files for *C. japonicus* Ueda107 (ASM1922v1) and *C. pinensis* DSM2588 (ASM2400v1) were obtained from ENSEMBLE, while that for *S. marcescens* PIC3611 was retrieved from NCBI Refseq (ASM2260299v1). Due to the differences in reference genome sources, the *S. marcescens* files required a parameter change in the htseq-count “feature type” from “gene” to “exon.” Differential gene expression using DESeq2 compared exponential growth of the experimental condition (either high or low melanin necromass) to that of glucose exponential growth, necromass stationary phase to that of glucose stationary phase, and necromass exponential growth to necromass stationary phase (38).

### Phenotypic microarray assay

Carbon and nitrogen utilization assays were completed by BioLog (Newark, DE) on a fee-for-service basis. Experiments were performed in biological triplicate in MOPS-defined media at 30°C for 24 h in a 96-well assay plate format using methods similar to those previously published (39, 40). A comprehensive list of all carbon and nitrogen sources tested can be found in Tables S24 to S26.

### Thermochemolysis-gas chromatography-mass spectrometry

*H. bicolor* necromass with an intermediate level of melaninization underwent thermochemolysis-GCMS (pyGCMS) to assess its biological makeup as previously described (23). Briefly, ca. 15 mL of tetramethylammonium hydroxide (TMAH) was added to 140–150 µg of ground and dried necromass. Samples were heated to 300°C at a rate of 720°C/min in a Gerstel Thermal Desorption Unit and immediately introduced into the GC column (HP-5MS, 30 m × 0.250 mm, 0.25 mm film thickness). The GC-oven (Agilent Technologies, 7890B, Santa Clara, CA, USA) was heated from 50°C to 320°C over 55 min and held at 320°C for 10 min. Molecules were ionized in an Agilent Technologies 5977A mass spectrometer by electron ionization with a voltage of 70 eV. The generated peaks were classified as aromatics, carbohydrates, lipids, nitrogen-containing, sterols, or compounds of unspecified origin by their mass spectra using Agilent ChemStation software (standard runs) and the NIST library. The N-containing fragments included proteins and amino sugars and, therefore, include N-containing chitin fragments. Single ion monitoring (SIM) was performed by Agilent MassHunter software, and the relative abundances of each compound were calculated using a MATLAB script.

## RESULTS

### Growth analyses

The three bacterial species were first grown on glucose, where minor growth variances were observed (Fig. S1**;** Table S1). *C. japonicus* had the fastest growth rate, shortest lag phase, and highest final cell density when provided glucose as the sole carbon source. Alternatively, *C. pinensis* and *S. marcescens* shared similar growth rates, with the former reaching a higher final cell density.

**Fig 1:**
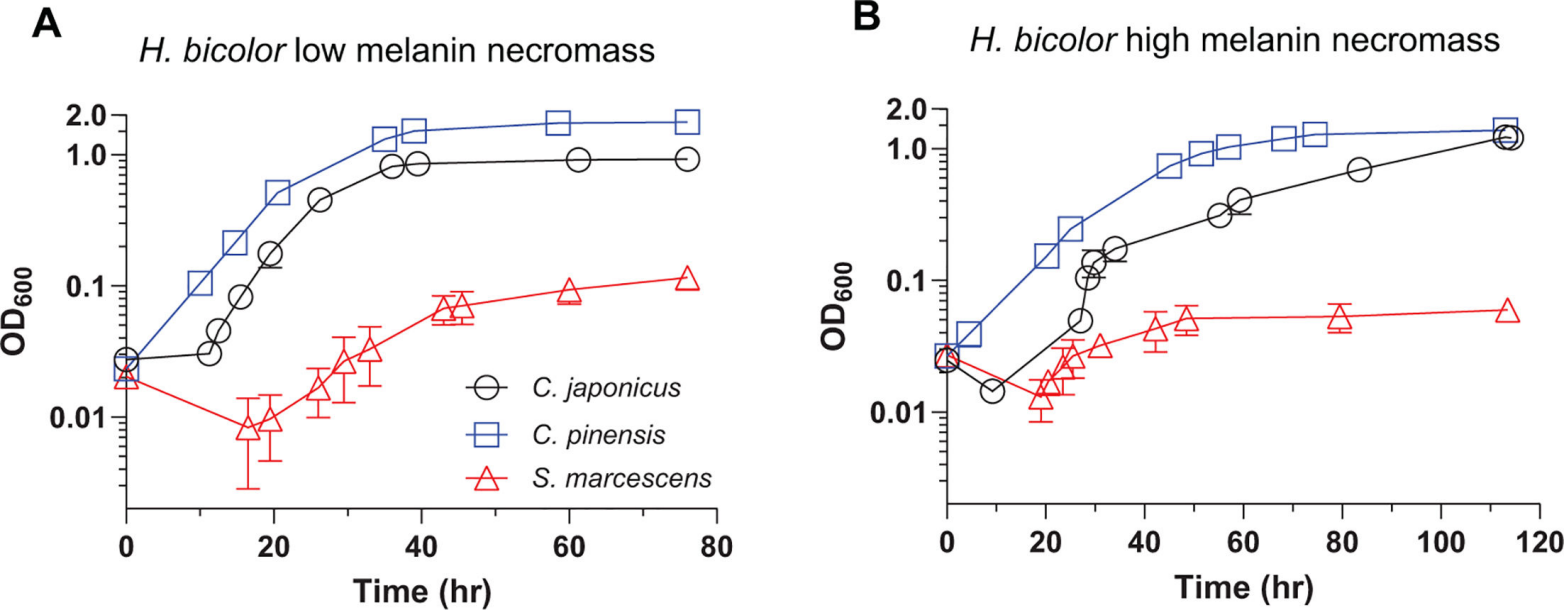
Growth analyses of *C. japonicus, C. pinensis,* and *S. marcescens* when provided MOPS minimal media supplemented with either (**A**) 1% (wt/vol) low melanin *H. bicolor* necromass or (**B**) 1% (wt/vol) high melanin *H. bicolor* necromass as the sole carbon sources. All growth experiments were completed in biological triplicate, with error bars representing standard deviations, although some are too small to be observed. Growth analyses were measured using test tubes and a spectrophotometer (Milton Roy Spec20D+). It should be noted that the growth analyses for each strain were conducted on different days, and timepoints were taken at different intervals in order to best capture the growth dynamics of each strain on both substrates. Such data were overlayed on one graph for each substrate to compare growth dynamics between species.

All three species were next grown on high and low melanin necromass to assess the growth variances between the two substrates and relative to a glucose growth baseline. *C. pinensis* had the shortest lag phase among the three species when growing on necromass (Fig. 1; Table S2), but the precise length of time in the lag phase could not be determined for *C. pinensis* on low melanin necromass due to its fast growth and gaps in sampling due to the design for protracted growth experiments. *C. pinensis* also had the highest final cell density on low melanin necromass and shared the highest final cell density on high melanin necromass compared to *S. marcescens* and *C. japonicus*. In a comparison of the growth dynamics on both necromass types, *C. pinensis* grew nearly twice as fast on the low than high melanin necromass and reached a higher cell density.

*C. japonicus* had a relatively consistent growth rate on low melanin necromass compared to high melanin necromass (Fig. 1; Table S2). Intriguingly, the growth dynamics of *C. japonicus* on high melanin necromass had several irregular phases of growth. Specifically, the exponential growth phase was rapid and only spanned roughly 5 h (OD_600_ 0.03–0.2), while the transition into the stationary phase lasted roughly 75 h (OD_600_ 0.2–1.0). Furthermore, the stationary phase was reached much sooner on the low melanin necromass at 40 h as opposed to the 100+ h on high melanin necromass.

Of the three species, *S. marcescens* exhibited the poorest growth on both necromass types, with comparatively very low final cell densities (Fig. 1; Table S2). However, the growth of *S. marcescens* on low melanin necromass had a final cell density that was double the final cell density on high melanin necromass. *S. marcescens* also maintained exponential growth on low melanin necromass for twice the time compared to high melanin necromass*,* albeit with a slower growth rate.

### Transcriptomic analyses—carbon and nitrogen utilization

#### Baseline gene expression profiles using glucose

Before conducting gene expression analyses for all three species on necromass, we identified genes with growth rate-controlled expression by comparing the RNAseq data of each strain grown on glucose during exponential growth compared to the stationary phase. The stationary phase was used as the reference condition for this analysis to maintain focus on the response of metabolically active cells. Since this type of analysis has already been reported in *C. japonicus* (41, 42)*,* our assessment initially focused on *C. pinensis* and *S. marcescens*. The comparative expression data are reported here as “Substrate GrowthPhase vs Substrate GrowthPhase” (e.g., Glc EXP vs Glc STA). These abbreviations indicate that the gene expression data are reflective of the former condition (e.g., Glc EXP) when compared to the latter condition (e.g., Glc STA). The supplementary tables containing differential gene expression data were filtered to only include the expression values when the *P*-value was significant at a level of <0.01. Therefore, genes that are absent from some tables (but not others) are due to removal because of a non-significant *P*-value. The predicted activity of CAZyme-encoding genes in *C. pinensis* DSM2588 was determined based on the annotated genome from EnsemblBacteria and previous proteomic studies (43). The predicted CAZyme-encoding genes for *S. marcescens* PIC3611 were determined by the annotated genome from NCBI (44). In total, *C. pinensis* has 357 CAZyme-encoding genes, while *S. marcescens* has 113.

**Fig 2:**
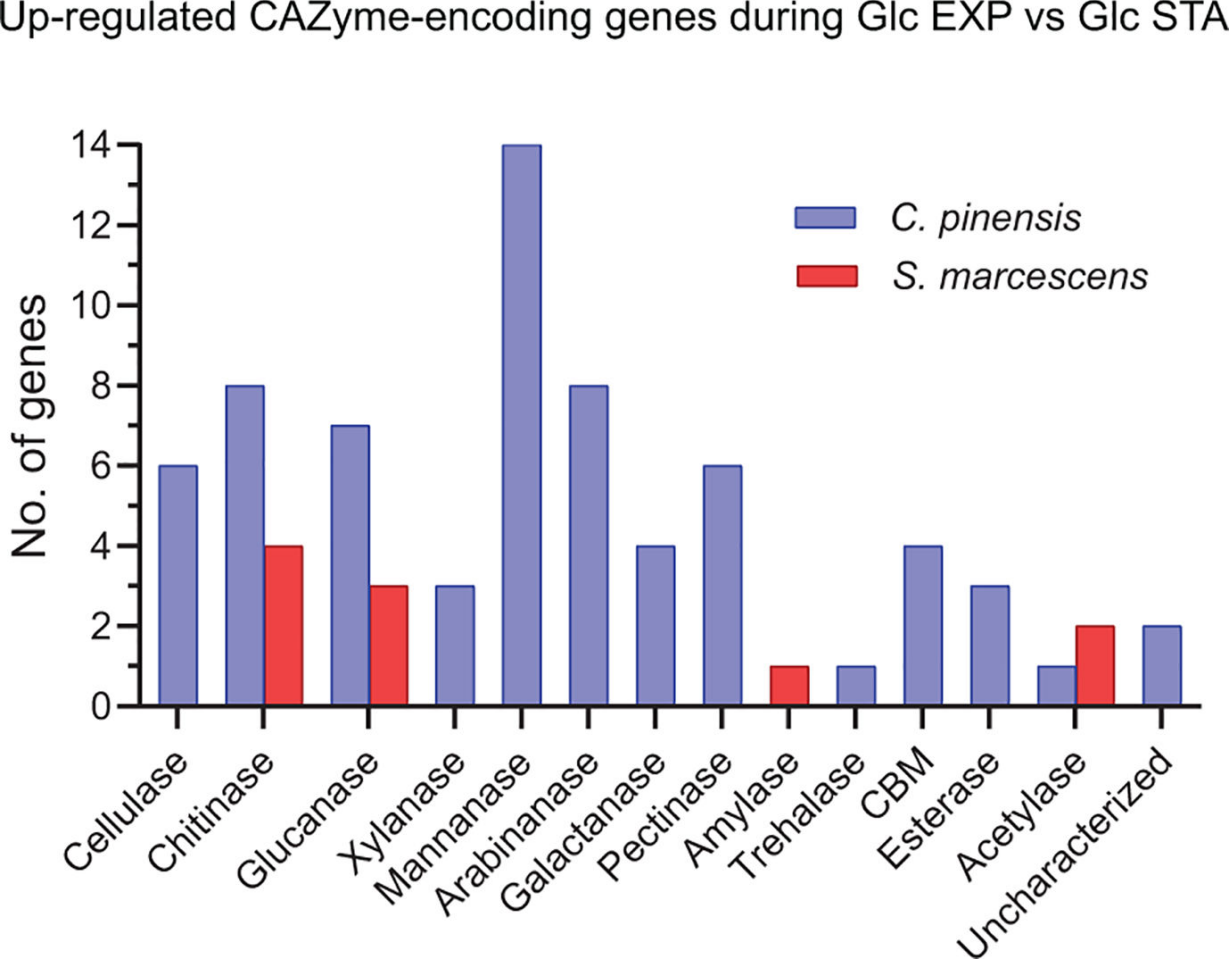
Upregulated *C. pinensis* and *S. marcescens* CAZyme-encoding genes grown on glucose during exponential growth compared to the stationary phase. Upregulated *C. pinensis* CAZyme-encoding genes are displayed in blue and those relating to *S. marcescens* are displayed in red. Genes which encode carbohydrate-binding modules are abbreviated as CBM.

In *C. pinensis*, a comparison of the gene expression response on glucose during exponential growth compared to the stationary phase showed upregulation of more than 2,000 genes (27% of the genome). The top 50 upregulated genes predominantly encoded proteins with functions in nutrient acquisition. This included genes which encode a variety of predicted functions such as transport, carbohydrate binding, glycoside hydrolysis, and nitrogen metabolism (Table S3). In terms of nitrogen utilization, the up-regulated genes encoded a glutamate-ammonia ligase (*cpin_7211*), glutamate synthetase (*cpin_1662*), threonine dehydratase (*cpin_1924*), histidine ammonia-lyase (*cpin_1853*), and two glutamate synthases (*cpin_0731* and *cpin_0730*). Additionally, among the upregulated genes were 67 predicted CAZyme-encoding genes (19% of total in genome). While all the listed CAZyme-encoding genes for *C. pinensis* are computationally predicted, not all have experimentally confirmed activities. The predicted gene products were diverse in putative activity and included mannanases, chitinases, arabinanases, cellulases, and pectinases (Fig. 2). The predicted CAZyme-encoding genes that exhibited the highest fold-change in expression during exponential growth (among the top 50 upregulated genes; ≥3.5-fold log_2_) were three glucanase-encoding genes (*cpin_6735, cpin_6736,* and *cpin_6737*), one chitinase-encoding gene (*cpin_5260*), and one α-mannanase-encoding gene (*cpin_4822*). The three glucanase-encoding genes are part of a predicted polysaccharide utilization locus (PUL), wherein *cpin_6737* has a four base-pair overlap with *cpin_6736,* and *cpin_6735* is 22 base pairs down-stream from *cpin_6736* (45). Additionally, *cpin_4822* is predicted to be part of a different PUL; however, the neighboring α-mannanase-encoding gene (*cpin_4820*) was far less expressed.

In *S. marcescens,* a comparison between growth phases on glucose elicited upregulation of more than 1,400 genes (26% of the genome). Among the top 50 up-regulated genes were products that largely confer motility, transport, and response regulation (Table S4). In terms of nitrogen utilization, *S. marcescens* elicited upregulation of seven genes during exponential growth on glucose. The genes and their predicted products included the small subunit of nitrite reductase (*L8N14_21965*), a nitrogen regulation protein NR(I) (*L8N14_23630*), glutamine synthetase (*L8N14_12535*)*,* glutamine amidotransferase (*L8N14_00130*), carbon-nitrogen hydrolase (*L8N14_05810*)*,* aspartate ammonia-lyase (*L8N14_17335*), and threonine ammonia-lyase (*L8N14_21270*). During growth on glucose, *S. marcescens* upregulated few computationally predicted CAZyme-encoding genes. Only 10 differentially expressed CAZyme-encoding genes (9% of total in genome) were identified and included glucanases, chitinases, carbohydrate deacetylases, and an amylase (Fig. 2). The most upregulated CAZyme-encoding genes (among the top 50 upregulated genes; >2-fold log_2_) encoded a polysaccharide deacetylase, two chitinases, phospho-β-glucosidase, and polysaccharide deacetylase with 3.2-, 3.1-, 2.7-, 2.4-, and 2.0-fold expression change (log_2_), respectively.

While *C. japonicus* analysis of CAZyme-encoding gene expression during exponential growth using glucose vs stationary phase was previously conducted, the upregulated nitrogen utilization-encoding genes were not discussed. Our analysis of those previously published data identified the upregulation of six nitrogen utilization-encoding genes that included glutamate dehydrogenase (*gdhA*), glutamine synthetase (*glnA*), glutamate synthase (*gltD*), threonine-ammonia lyase (*ilvA*), glutamine-dependent NAD + synthetase (*adgA*), and nitroreductase (*cja_1559*).

#### CAZyme gene expression during growth on high and low melanin necromass

As a complement to the growth analyses, we conducted a transcriptomic study to better characterize bacterial *H. bicolor* decomposition. All RNAseq data underwent differential gene expression analysis using comparisons to glucose or opposing growth phases to identify physiologically relevant CAZyme-encoding genes, an approach that has been done previously (15, 16). The primary observation made from these results was that CAZyme-encoding gene expression for all three bacteria was almost identical for both types of necromass (Tables S5 to S22). Given that low melanin necromass possesses greater proportions of carbohydrates than high melanin necromass (23), below we report the results for CAZyme-encoding gene expression only for that necromass type. However, graphical representations of CAZyme-encoding gene expression for each species on high melanin necromass are presented in Fig. S2. Notably, several of the discussed CAZyme-encoding genes are only computationally predicted and not yet biochemically confirmed. CAZyme-encoding genes that are described as “uncharacterized” are, therefore, predicted hydrolases that have not been classified into a specific family of glycoside hydrolase or esterase.

#### *C. pinensis* CAZyme-encoding gene expression is regulated both temporally (exponential vs stationary phase) and by substrate (necromass vs glucose)

**Fig 3:**
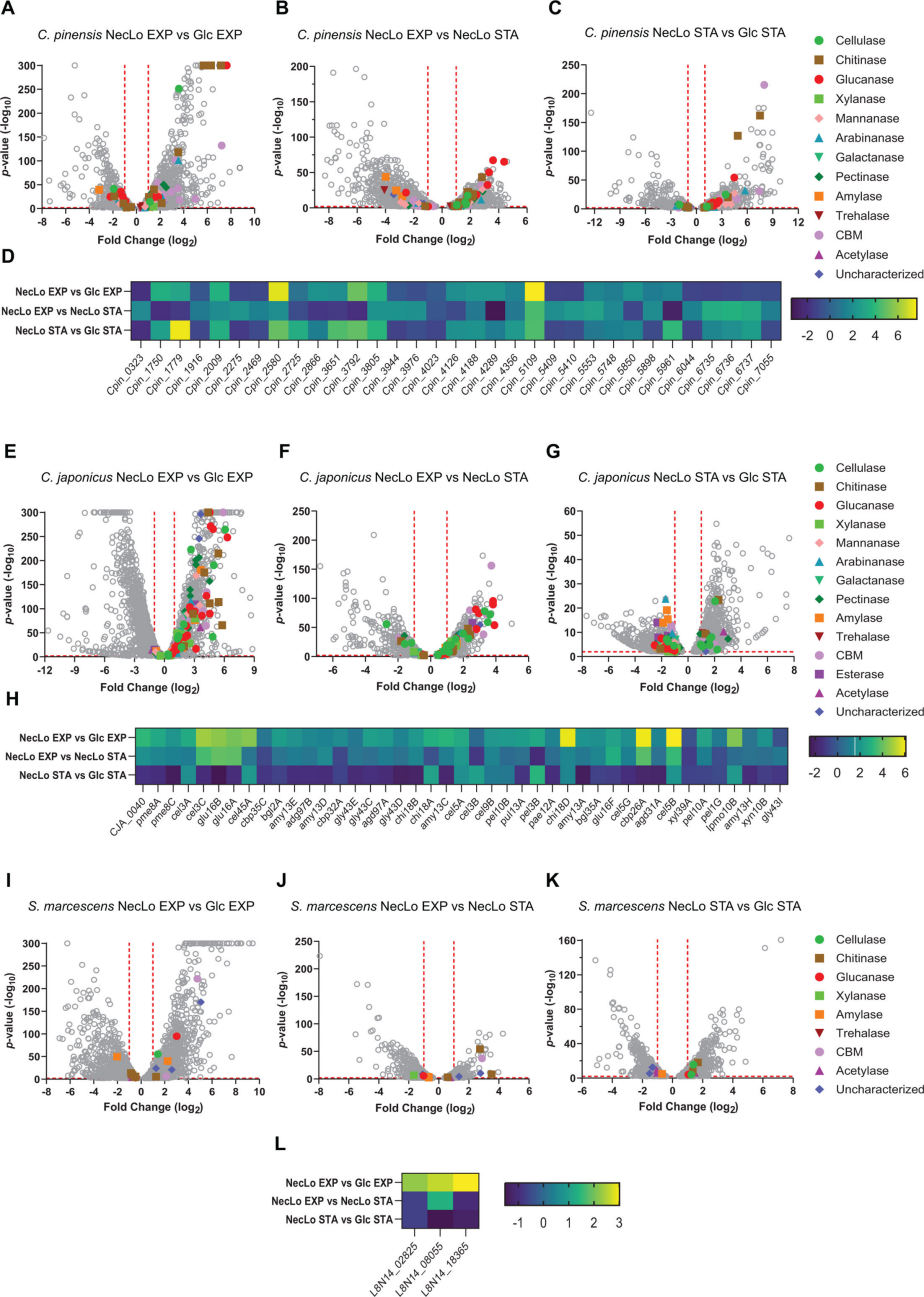
Changes to CAZyme-encoding gene expression during various growth phases on low melanin necromass. (**A**) Volcano plot representation of gene expression data for *C. pinensis* during exponential growth on low melanin necromass compared to glucose. (**B**) Volcano plot representation of gene expression data for *C. pinensis* grown on low melanin necromass during exponential growth compared to the stationary phase. (**C**) Volcano plot representation of gene expression data for *C. pinensis* during the stationary phase on low melanin necromass compared to glucose. (**D**) Heat map showing changes in differential CAZyme gene expression in *C. pinensis*. (**E**) Volcano plot representation of gene expression data for *C. japonicus* during exponential growth on low melanin necromass compared to glucose. (**F**) Volcano plot representation of gene expression data for *C. japonicus* grown on low melanin necromass during exponential growth compared to the stationary phase. (**G**) Volcano plot representation of gene expression data for *C. japonicus* during the stationary phase on low melanin necromass compared to glucose. (**H**) Heat map showing changes in differential CAZyme gene expression in *C. japonicus*. (**I**) Volcano plot representation of gene expression data for *S. marcescens* during exponential growth on low melanin necromass compared to glucose. (**J**) Volcano plot representation of gene expression data for *S. marcescens* grown on low melanin necromass during exponential growth compared to the stationary phase. (**K**) Volcano plot representation of gene expression data for *S. marcescens* during the stationary phase on low melanin necromass compared to glucose. (**L**) Heat map showing changes in differential CAZyme gene expression in *S. marcescens*. Open gray circles in the volcano plots represent a single gene, and colored symbols represent a CAZyme-encoding gene that correlates to the symbol/color provided in the legend.

Our *C. pinensis* transcriptome analysis during growth on glucose in exponential growth compared to the stationary phase found that 33% of *C. pinensis* CAZyme-encoding genes were regulated temporally. Comparison of the transcriptomic data for exponential growth on low melanin necromass compared to glucose (NecLo EXP vs Glc EXP) also identified CAZyme-encoding genes with substrate-induced expression. This latter comparison identified 1,150 significantly upregulated genes, with 41 being CAZyme-encoding genes (*P*-value < 0.01) (Fig. 3A). The upregulated CAZyme-encoding genes were diverse in predicted activity, encompassing 10 putative carbohydrate-binding modules (CBMs), eight pectinases, seven chitinases, four arabinanases, three glucanases, three uncharacterized CAZymes, two mannanases, two cellulases, one xylanase, and one amylase. Among the top 50 upregulated genes (>5.0-fold log_2_), the functional activities observed were for gluconeogenesis, nutrient transport, and carbohydrate degradation (Table S5). The CAZyme-encoding genes found within the top 50 up-regulated genes included three chitinase-encoding genes (*cpin_2580, cpin_2186,* and *cpin_2184*), one CBM-encoding gene with an encoded protein belonging to GH16 (*cpin_2187*), one putative CBM-containing gene (*cpin_3792*), and one glucanase-encoding gene (*cpin_5109*) (Fig. 3D). Notably, *cpin_2184, cpin_2186,* and *cpin_2187* belong to a fungal cell wall utilization locus (FCWUL) and were recently renamed as *chiA, chiB,* and *glu16A,* respectively (46).

Gene expression comparing growth on necromass during the exponential and stationary phases (NecLo EXP vs NecLo STA) found up-regulation of 1,361 genes during exponential growth, with 46 CAZyme-encoding genes (*P*-value < 0.01) (Fig. 3B). Among the 46 up-regulated CAZyme-encoding genes included 10 chitinases, nine pectinases, six putative CBMs (four of which contain GH2 domains), five glucanases, five mannanases, five cellulases, four arabinanases, two galactanases, and one uncharacterized CAZyme. Unsurprisingly, the top 50 up-regulated genes (> 3.4-fold log_2_) identified genes encoding proteins important for cellular metabolism and growth. Gene products included cobalamin synthesis, glycoside hydrolysis, cellular growth, and nutrient transport (Table S6). The CAZyme-encoding genes identified within the top 50 up-regulated genes included two β-glucanase (*cpin_5109* and *cpin_6736*) (Fig. 3D). Differential expression analysis on glucose found that *cpin_6736* gene expression was induced by the growth rate and was part of a putative β-glucan utilization operon. Conversely, *cpin_5109* was not in an operon and its expression was induced by both the growth rate and substrate presence, with stronger up-regulation in the presence of *H. bicolor* necromass.

The final differential expression analysis for *C. pinensis* compared the stationary phase on low melanin necromass to the stationary phase on glucose (NecLo STA vs Glc STA). Under these conditions, there were 1,092 up-regulated genes with 34 being CAZyme-encoding genes (*P*-value < 0.01) (Fig. 3C). In terms of CAZyme-encoding gene expression, there were 34 up-regulated genes that included eight CBMs (one containing a GH16 domain, one with a GH64 domain, one with a GH87 domain, and two with GH2 domains), six glucanases, six mannanases, five chitinases, three arabinanases, two pectinases, two cellulases, one xylanase, and one uncharacterized CAZyme. The top 50 up-regulated genes (>6.0-fold log_2_) encoded activities relative to bacterial respiration, TonB-dependent transport, and carbohydrate metabolism (Table S7). The CAZyme genes that were up-regulated more than 6.0-fold (log_2_) were *cpin_2184* (*chiA*)*, cpin_2186* (*chiB*)*, cpin_2187* (*glu16A*)*,* and *cpin_1779* (Fig. 3D). As noted in the NecLo EXP vs Glc EXP comparison, *cpin_2184–cpin_2187* belong to the recently characterized FCWUL.

#### *C. japonicus* has complex CAZyme-encoding gene expression during growth on necromass

Comparison of transcriptomic data collected during exponential growth on low melanin necromass compared to glucose was the most informative evaluation for *C. japonicus* gene expression. There were 1,077 up-regulated genes (*P*-value < 0.01) with 100 being CAZyme-encoding genes which included 19 glucanases, 15 pectinases, 9 cellulases, 9 CBMs (two of which contain an AA10 domains), 8 chitinases, 7 amylases, 6 xylanases, 6 galactanases, 8 arabinanases, 4 mannanases, 4 uncharacterized CAZymes, 3 acetylases, and 2 esterases (Fig. 3E). A complex gene expression response was not unsurprising for *C. japonicus* based on previous RNAseq studies (47), and 19 up-regulated glucanase-encoding genes would be expected based on glucans being the dominant polysaccharide found in *H. bicolor* cell walls. The top 50 up-regulated genes (>5.3-fold log_2_) encoded activities for ribosomal RNA, electron transport, and carbohydrate metabolism (Table S11). This list also included CAZyme-encoding genes *glu81A* (glucanase)*, cel5B* (cellulase)*, cbp26A* (CBM)*, chi18D* (chitinase)*, csn46F* (chitinosanase)*,* and *chi19A* (chitinase) (Fig. 3H). Notably, a *C. japonicus* Δ*chi18D* mutant has previously been shown to be essential to *C. japonicus* α-chitin degradation, while Δ*chi19A* and Δ*csn46F* exhibited minor growth rate defects (48, 49).

*C. japonicus* growth on low melanin necromass during exponential growth compared to the stationary phase had far fewer up-regulated CAZyme-encoding genes. In total, there were 548 up-regulated genes with 51 being CAZyme-encoding genes (*P*-value < 0.01) (Fig. 3F). The 51 up-regulated CAZyme genes included 8 glucanases, 8 cellulases, 5 chitinases, 5 CBMs, 4 pectinases, 3 uncharacterized CAZymes, 3 esterases, 3 amylases, 2 xylanases, 2 arabinanases, 1 mannanase, and 1 galactanase. While fewer CAZyme-encoding genes were up-regulated, one-third of them were among the most up-regulated genes (top 50; >2.4-fold log_2_). The remaining genes expressed more than 2.4-fold (log_2_) encoded TonB-dependent transporters, cell wall biosynthesis proteins, and motility proteins (Table S12). The highly up-regulated CAZyme-encoding genes were *glu16B, glu81A, glu16A, cbp26A, cel6A, cel3C, cbp6B, cel5B, cbp2E, gly30A, glu5A, glu16F, pul13B, cbp2D, ce2C,* and *csn46F*, respectively (Fig. 3H). The high proportion of glucanase-encoding genes observed in this comparison provides ample targets for future physiological studies of *C. japonicus* CAZyme using gene deletion analyses during the degradation of fungal glucans.

Comparing the stationary phase on low melanin necromass with the stationary phase on glucose suggested some nutrient acquisition was still occurring in *C. japonicus* cells on low melanin necromass. There were 660 up-regulated genes with 17 being CAZyme-encoding genes (*P*-value < 0.01) (Fig. 3G). The CAZyme-encoding genes included six cellulases, two chitinases, two CBMs (both of which contain AA10 domains), two pectinases, two uncharacterized CAZymes, one acetylase, and one trehalase. The top 50 up-regulated genes (>3.1-fold log_2_) encoded ribosomal RNA, nitrogen utilization proteins, and transposons (Table S13). Interestingly, this comparison lacked up-regulation of any CAZyme-encoding genes in the top 50 up-regulated genes. However, in the top 100 up-regulated genes (2.5-fold log_2_), there were two CAZyme-encoding genes (*pel3B* and *pda4B*)*,* which encode a pectate lyase and polysaccharide deacetylase, respectively (Fig. 3H).

#### *S. marcescens* exhibits a poor CAZyme-encoding gene expression response on necromass

Corresponding with the poor growth of *S. marcescens* on *H. bicolor* necromass, this species elicited a sparse CAZyme-encoding gene expression response on that substrate. *S. marcescens* cells collected during exponential growth on low melanin necromass compared to glucose resulted in up-regulation of 1,130 genes with only 9 encoding CAZymes (*P*-value < 0.01) (Fig. 3I). Among the up-regulated CAZyme-encoding genes included three uncharacterized CAZymes, two CBMs, one chitinase, one glucanase, one cellulase, and one amylase. The top 50 up-regulated genes (>5.2-fold log_2_) encoded activities for transport, amino acid metabolism, and stress response (Table S17). While there were no CAZyme-encoding genes among the top 50 up-regulated genes, there were two included in the top 100, which were for a lytic polysaccharide monooxygenase (*L8N14_14090*) and a CBM (*L8N14_01950*) (Fig. 3L).

Growth of *S. marcescens* on low melanin necromass during exponential growth compared to the stationary phase showed up-regulation of numerous genes involved in cell growth. There were 219 genes up-regulated with five CAZyme-encoding genes (*P*-value < 0.01) (Fig. 3J). Among the up-regulated CAZyme-encoding gene included two chitinases, two uncharacterized CAZymes, and one CBM. The top 50 up-regulated genes (>1.7-fold log_2_) encoded activities such as carbohydrate metabolism, transcriptional regulators, and transporters (Table S18). Unlike the exponential growth phase comparisons, here, the top 50 up-regulated genes included the CAZyme-encoding genes *L8N14_14080, L8N14_01950, L8N14_14090,* and *L8N14_01905*. These are predicted to encode a chitinase, CBM, lytic polysaccharide monooxygenase, and another chitinase, respectively (Fig. 3L).

Our final comparison comprised of *S. marcescens* during the stationary phase on low melanin necromass compared to glucose. Here, we observed up-regulation of 730 genes with 7 being CAZyme-encoding genes (*P*-value < 0.01) (Fig. 3K). The top 50 up-regulated genes (>2.7-fold log_2_) encoded activities for nutrient transport, ribosomal RNA, and cell wall repair (Table S19). However, far fewer CAZyme-encoding genes were up-regulated in these conditions. Only two chitinases, two glucanases, two cellulases, and one amylase were identified; however, none were among the top 50 or even top 100 up-regulated genes. In fact, the chitinase-encoding gene (*L8N14_22810*) was the most up-regulated CAZyme encoding gene with a 1.7-fold expression change (log_2_).

#### Gene expression of nitrogen utilization-encoding genes was highly variable across species grown on necromass

Previous reports have indicated that the high melanin type of *H. bicolor* has significantly less nitrogen than the low melanin type (23). Despite supplementation of ammonia in the growth medium, we still observed differences in the gene expression response of *S. marcescens* and *C. pinensis* under conditions which compared exponential growth on necromass vs glucose as well as the stationary phase on necromass vs glucose (Table S23). Similar to the CAZyme-encoding gene expression response, expression comparisons of nitrogen utilization-encoding genes were highly similar between the high and low necromass substrates (Tables S6, S9, S12, S15, S18, and S21). Interestingly, however, all three species up-regulated a different set of nitrogen utilization-encoding genes during exponential growth on glucose (Glc EXP vs Glc STA) than on necromass (Nec EXP vs Glc EXP) (Table S23). Considering this, we hypothesize that the up-regulated nitrogen utilization-encoding genes are likely important for nitrogen acquisition from necromass and are not solely reflective of growth rate-dependent gene expression.

During exponential growth (NecLo EXP vs Glc EXP), *C. pinensis* up-regulated four nitrogen utilization genes (*cpin_2006, cpin_3374, cpin_1695,* and *cpin_0230*) on low melanin necromass compared to glucose (Table S23; Table S5). Comparatively, only *cpin_1695* was also up-regulated during exponential growth on high melanin necromass compared to glucose (Table S8). During the stationary phase, *C. pinensis* on low melanin necromass elicited up-regulation of *cpin_4284*, *cpin_0230*, *cpin_1695*, and *cpin_5012* (Table S23; Table S7). The stationary phase *C. pinensis* response on high melanin necromass only up-regulated *cpin_0230* (Table S10). Interestingly, *C. pinensis* did not up-regulate any of the same nitrogen utilization-encoding genes on low melanin necromass compared to those during exponential growth on glucose. Instead, *C. pinensis* up-regulated genes that encoded nitroreductases, nitropropane dioxygenases, and nitrogen-fixing proteins when grown on necromass compared to glucose.

*S. marcescens* up-regulated the most nitrogen-utilization encoding genes on necromass compared to glucose. During exponential growth on low melanin necromass (NecLo EXP vs Glc EXP), nine *S*. *marcescens* nitrogen utilization-encoding genes were up-regulated, which included *L8N14_11925*, *L8N14_06450*, *L8N14_10750*, *L8N14_04185*, *L8N14_21470*, *L8N14_16415*, *L8N14_18380*, *L8N14_13885*, *L8N14_10745*, *L8N14_18910*, and *L8N14_15185* (Table S23; Table S17). On high melanin necromass (NecHi EXP vs Glc EXP), the up-regulated genes during exponential growth were *L8N14_11925, L8N14_06450,* and *L8N14_21470* (Table S20). During the stationary phase on low melanin necromass, *S. marcescens* up-regulated *L8N14_21965*, *L8N14_05810*, *L8N14_24000*, *L8N14_23630*, *L8N14_12535*, *L8N14_03515*, and *L8N14_18910* (Table S23; Table S19). During the stationary phase on high melanin necromass, the up-regulated genes were *L8N14_04185, L8N14_21965*, *L8N14_23630*, *L8N14_12535*, and *L8N14_05810* (Table S22). Overall, the only nitrogen utilization-encoding genes that were up-regulated in both the Glc EXP vs Glc STA and Nec STA vs Glc STA comparisons were four genes which encoded a nitrite reductase, carbon-nitrogen hydrolase, nitrogen regulation protein, and glutamine synthetase (Table S23).

During exponential growth on necromass compared to glucose (Nec EXP vs Glc EXP)*, C. japonicus* up-regulated seven nitrogen utilization genes (*cja_2161, ald, cja_3392, ntrC, norB, cja_3390, nirB,* and *nirD*) (Table S23; Tables S11 and S14). During the stationary phase on low melanin necromass compared to glucose (Nec STA vs Glc STA), *C. japonicus* up-regulated nine nitrogen utilization genes that included *cja_3392*, *glnA*, *ntrC*, *nirB*, *nirD*, *cja_3390*, *cja_3536*, *gltD*, and *gltB* (Table S23; Table S13). Comparatively, the stationary phase on high melanin necromass up-regulated the same set of genes, although *cja_3536* was absent and *cja_1973* was present, which encodes the α subunit of glutamate synthase (Table S16). Among the up-regulated nitrogen utilization-encoding genes on necromass*,* the only genes that were also up-regulated on glucose (Glc EXP vs Glc STA) were expressed during the stationary phase on necromass. These encoded a glutamine synthetase (*glnA*) and glutamate synthase (*gltD*).

### Carbon and nitrogen utilization analyses

**Fig 4:**
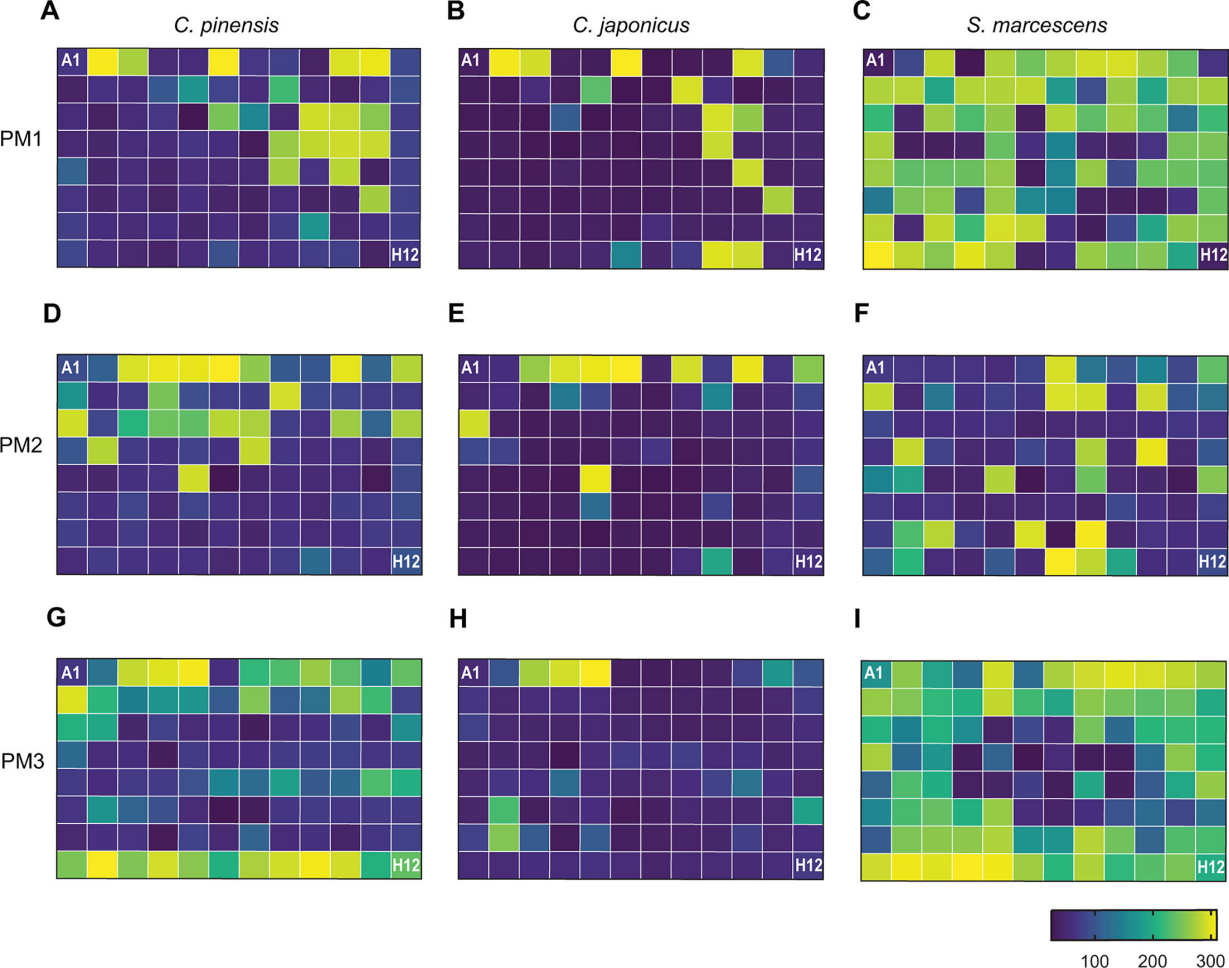
Phenotypic microarray assay of *C. japonicus, C. pinensis,* and *S. marcescens* metabolic activity on various carbon and nitrogen sources. Panels A, D, and G correspond to *C. pinensis* activity. Panels B, E, and H correspond to *C. japonicus* activity. Panels C, F, and I correspond to *S. marcescens* activity. Carbon and nitrogen sources for each well are listed in Tables S24 to 26 where Table S24 corresponds to PM1 in panels A–C, Table S25 corresponds to PM2 in panels D–F, and Table S26 corresponds to PM3 in panels G–I. Metabolic activity was determined using dye reduction kinetic curves from an OmniLog where activity is positively correlated with a brighter color.

To better characterize the metabolic potential of *C. pinensis, C. japonicus,* and *S. marcescens*, we assessed the metabolic phenotypes of the three species on a broad range of carbon and nitrogen sources (Fig. 4; Tables S24 to 26). These assays yielded multiple intriguing results, for example, the poor metabolic activity of *C. pinensis* on glucose. As demonstrated in Fig. S1, this strain can grow well on glucose as a sole carbon source. These differences may be a consequence of the BioLog system, which assesses bacterial metabolic respiration and not growth. Moreover, the BioLog system has a universal bacterial assay design; therefore, it is possible that the tested substrates could be better optimized for utilization studies. Despite these limitations, the BioLog experiments were able to assay a broad range of substrates and further inform our models for carbon and nitrogen metabolism of the three bacterial species they pertain to necromass utilization.

The poor metabolic activity of *C. pinensis* on α-mannan was a surprising phenotype considering the strain has 10 predicted α-mannanases (45). However, D-mannose was the only relevant monosaccharide that elicited high metabolic activity by *C. pinensis* (Fig. 4A, D and G). The strain also exhibited high metabolic activity on glucosamine and *N*-acetylglucosamine compared to the mannose- and galactose-based amino sugars. Additionally, *C. pinensis* could utilize L-amino acids as sole nitrogen sources but had poor metabolic activity when provided with the same substrates as sole carbon sources, with one exception being L-aspartic acid. Overall, *C. pinensis* was most metabolically active on nitrite, nitrate, and urea.

In addition to the poor metabolic activity of *C. japonicus* on α-mannan, the strain also had poor activity on D-mannose, as has been previously recorded (47, 50). Alternatively, *C. japonicus* exhibited high metabolic activity on D-galactose and D-glucose. This was also true on the glucose-based amino sugars glucosamine and *N-*acetylglucosamine when utilized as carbon sources (Fig. 4B, E and H). A lack of metabolic activity by *C. japonicus* was observed on L-amino acids as either carbon or nitrogen sources. In fact, the only nitrogen sources that *C. japonicus* exhibited high metabolic activity on were nitrite, nitrate, and urea.

*S. marcescens* had the broadest metabolic range of the three strains on carbon and nitrogen sources assayed. *S. marcescens* had very high activity on D-glucose, D-galactose, D-mannose, and the amino sugars, with the sole exception being *N*-acetyl mannosamine (Fig. 4C, F and I). The amino sugars also provided *S. marcescens* with a small amount of activity when provided as a sole nitrogen source. When grown on L-amino acids as either carbon or nitrogen sources, *S. marcescens* was highly metabolically active. Activity was highest on the L-amino acids when the strain utilized them for nitrogen compared to carbon. Finally, testing *S. marcescens* metabolic activity on canonical nitrogen sources (nitrite, nitrate, ammonia, urea) indicated all four could be utilized, with ammonia and urea eliciting the highest activity.

## DISCUSSION

The decomposition of fungal necromass has been shown to significantly contribute to the both carbon and nitrogen cycling in forest soils (51). Therefore, understanding the mechanisms of its decay and the saccharifying properties of its decomposers is important to understand the dynamics of soil nutrients, as recently reviewed (24). The use of *Hyaloscypha bicolor* as a model necromass benefits this area of research through its potential to exist in chemically distinct but genetically equivalent phenotypes. Previous chemical analysis of the *H. bicolor* cell wall has shown that the low melanin type contains twice the carbohydrates as the high melanin type, making carbohydrates roughly 40% of the low melanin *H. bicolor* cell wall (23). Interestingly, despite the difference in polysaccharide proportions between the two types of *H. bicolor,* the CAZyme-encoding gene expression responses of all three bacteria were almost identical on both necromass types (Fig. 3; Fig. S2). While the exact concentrations of each polysaccharide in the *H. bicolor* cell wall are still unknown, other fungal species belonging to the phylum *Ascomycota* (e.g., *Yarrowia lipolytica, Aspergillus fumigatus,* and *Candida albicans*) had glycan content ranging between 45%–60% glucans and 16%–20% chitin (52–54). Our pyGCMS analysis of *H. bicolor* revealed a somewhat lower glycan content (~29% carbohydrate) and chitin content (~13% of N-containing compounds, which includes proteins and amino sugars like chitin) (Table S27). Future work parsing amino sugars from amino acids and determining the glucan content of *H. bicolor* cell walls with different levels of melanization will facilitate more accurate comparisons to other fungi.

The signals that elicit the observed CAZyme-encoding gene expression responses are clearly similar, suggesting that melanization does not impact bacterial carbon utilization gene expression, at least under the assay conditions used. However, these necromass variants are particularly interesting when considering the notable differences in gene expression of the nitrogen utilization-encoding genes of *C. pinensis* and *S. marcescens* on high vs low melanin necromass (Table S23). Collectively, these results complement *H. bicolor* decomposition studies, wherein nitrogen appears to be a substantial factor to its degradation (18, 21, 22, 55).

### *Chitinophaga pinensis* is an early-stage necromass degrader with metabolic potential to persist into later stages

*C. pinensis* exhibited the quickest transition to exponential growth on both types of necromass (Fig. 1). This is likely due to its growth-rate dependent CAZyme-encoding gene expression response since neither of the other strains elicited a response quite as broad as *C. pinensis* during exponential growth on glucose compared to the stationary phase. The up-regulated CAZyme-encoding genes in this growth comparison overwhelmingly encoded mannanase gene products, followed by cellulases, chitinases, glucanases, and arabinanases (Fig. 2). Notably, up-regulation of chitinase-, glucanase-, and mannanase-encoding genes upon entrance into exponential growth logically increases the adaptability of *C. pinensis* to grow on fungal necromass. This observed specialty of *C. pinensis* to quickly adapt to its substrate was partly conveyed *in situ*. A study on bacterial genera presence during various phases of *H. bicolor* decomposition showed *Chitinophaga* was among the dominant genera during the early- and mid-stages of decay (21). Interestingly, however, *Chitinophaga* has also been identified as the dominating genus during late-stage degradation of necromass associated with plant hosts (21), which could be either due to flexible metabolic capacity or potentially the decomposition of newly senescent microbes that were themselves a part of the early necromass decomposition community.

**Fig 5:**
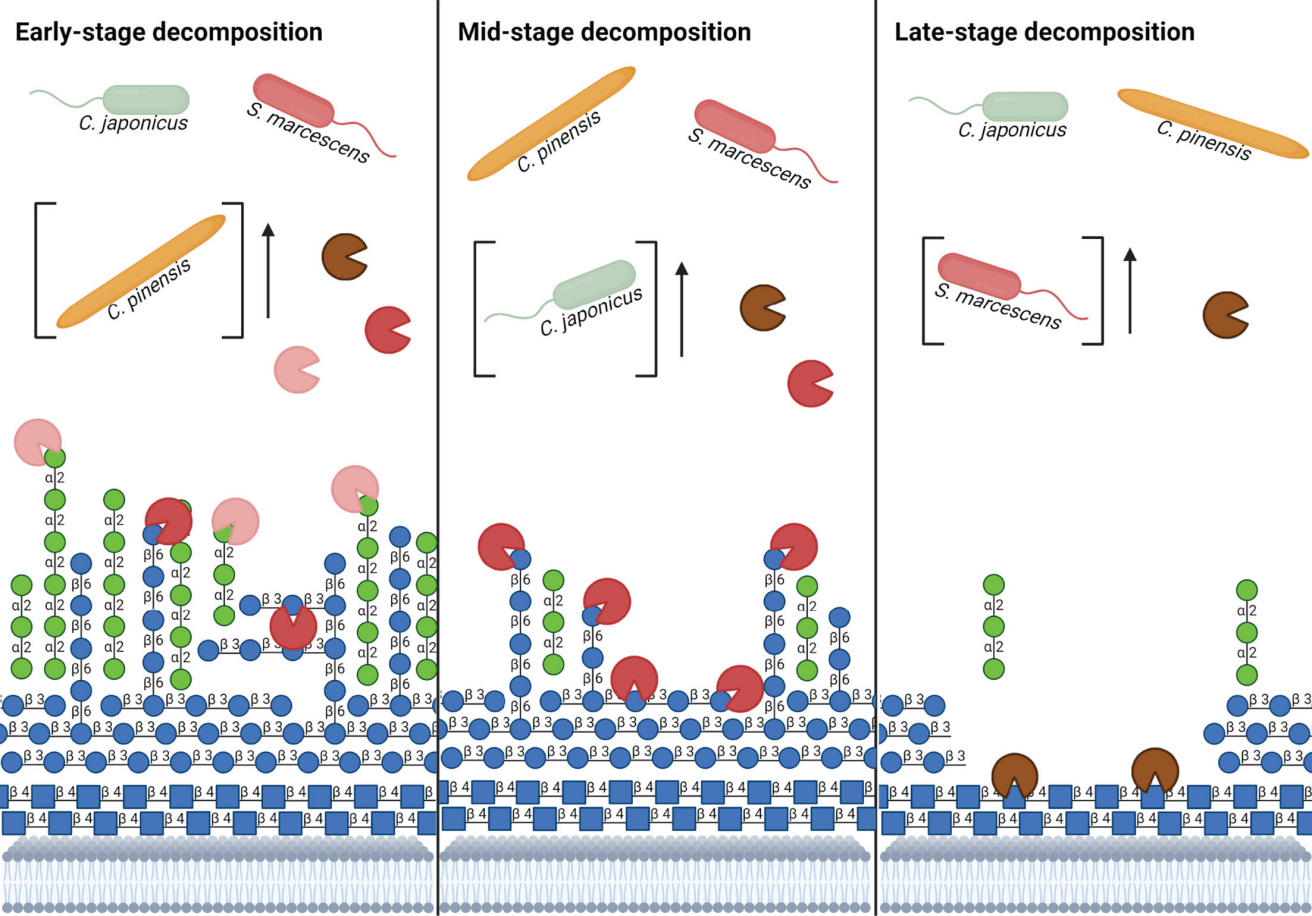
Model of necromass decomposition by *C. pinensis, C. japonicus,* and *S. marcescens. C. pinensis* is hypothesized to be the early-stage degrader due to its growth-rate dependent expression of CAZyme-encoding genes. *C. pinensis* is also the only species among the three with mannanase-encoding genes. *C. japonicus* is the likely mid-stage degrader with presence throughout the degradation process due to its abundance of glucanase- and chitinase-encoding genes. Finally, *S. marcescens* is depicted as the late-stage degrader since it is exceptionally efficient at utilizing Chitin as a carbon and nitrogen source. The model of necromass cell wall is simplified to only show the most common polysaccharides. Chitin is depicted in the chains of blue squares, glucans are shown as the blue circles, and mannans are shown in the green circles. In terms of the represented CAZymes, mannanases are pink, glucanases are red, and chitinases are brown.

Despite the broad CAZyme-encoding gene expression response of *C. pinensis* during exponential growth, this species had a more specific response when grown on necromass (Fig. 3A through D). In the NecLo EXP vs Glc EXP comparison, the chitinase-, glucanase-, and CBM-encoding genes were among the most up-regulated. However, one of the more revealing comparisons was in NecLo EXP vs NecLo STA and NecLo STA vs Glc STA. There, the NecLo EXP vs NecLo STA comparison overwhelmingly encoded β-glucanases. This varied from the NecLo STA vs Glc STA comparison which up-regulated more chitinase-encoding genes. This adjustment to the expression response could be reflective of polysaccharide presence, wherein the outer β-glucan layer of *H. bicolor* has been deconstructed enough to reveal the chitinous inner layer of the substrate. Alternatively, increases in chitinase gene expression during the stationary phase could instead be an attempt at nitrogen acquisition since the strain can utilize *N*-acetylglucosamine and glucosamine as nitrogen sources (Fig. 4). Moreover, the combined broad expression of CAZyme-encoding genes during exponential growth paired with substrate-specific induced expression of β-glucanases contributes to our hypothesis that *C. pinensis* would prevail as the early-stage degrader among the three species (Fig. 5). Two polysaccharide utilization loci (PUL) appeared to be most prevalently expressed. These PULs encompass *cpin_6730 – cpin_6742* and *cpin_2184 – cpin_2192*. The former was up-regulated during exponential growth and encodes for an endo-β-1,3-glucanase, endo-β-1,6-glucanase, and an exo-β-glucosidase (56). The latter PUL belongs to a recently identified and characterized fungal cell wall utilization locus (FCWUL) (46). The poor metabolic activity of *C. pinensis* when grown on α-mannan alone was a surprising result. Analysis of the predicted PULs in *C. pinensis* indicates nine of its α-mannanase-encoding genes lie within PULs; however, none of these PULs possess more than one α-mannanase-encoding gene or any other fungal cell wall-degrading CAZyme (57). Interestingly, *C. pinensis* has been shown to grow on plant β-mannans such as konjac glucomannan and carob galactomannan (45, 58), but there have been no growth studies of the bacterium on chitin.

*C. pinensis* exhibited changes in gene expression of nitrogen utilization-encoding genes on high vs low melanin necromass (Table S23). These differences were observed between the two necromass types and between the two growth phases. Exponential growth on low melanin vs high melanin necromass (Nec EXP vs Glc EXP) resulted in a higher number of up-regulated genes important to nitrogen acquisition from amino acids (*cpin_2006* and *cpin_3374*). This differed from the up-regulated nitrogen utilization-encoding genes during the stationary phase on low vs high melanin necromass, which up-regulated two nitroreductase-encoding genes (*cpin_4284* and *cpin_5012*). While little is known about nitrogen metabolism in *C. pinensis,* these results may indicate that a higher protein presence may exist in the cell wall of low melanin necromass from which *C. pinensis* can derive its nitrogen. An alternative hypothesis is that fungal proteins are better protected from degradation in high melanin cell walls, so the observed increase in gene expression under the low melanin conditions is because fungal proteins are more accessible.

### *C. japonicus* has exceptional efficiency during polysaccharide degradation as a mid-stage degrader of necromass

Growth on fungal cell wall degradation products showed *C. japonicus* is highly active on the canonical polysaccharide constituents (glucose, glucosamine, and *N*-acetylglucosamine), with little metabolic activity on other amino sugars (Fig. 4). Notably, *C. japonicus* had the fastest growth rates on both necromass types despite having a longer lag phase than *C. pinensis* (Table S2). Since *C. japonicus* has all the necessary CAZymes to completely degrade the fungal cell wall and has a faster growth rate on necromass, we suggest this species to be a strong candidate for a versatile mid-stage degrader of necromass (Fig. 5).

*C. japonicus* has an extensive history of transcriptomic and mutational analyses on various polysaccharides during different growth phases (42, 47, 49, 59). Notably, the CAZyme-encoding gene expression response of *C. japonicus* on low melanin *H. bicolor* necromass has thus far been the only substrate to elicit such strong up-regulation of non-cellulase, β-glucanase-encoding genes. The predicted glucanases largely belong to GH16 and GH81 which hydrolyze diverse sugar linkages and β-1,6-glucans, respectively (25, 60). Within the highly up-regulated CAZyme-encoding genes during exponential growth on necromass compared to glucose, the only strongly up-regulated β-glucanase-encoding gene was *glu81A*. However, the response of *C. japonicus* cells grown on *H. bicolor* during exponential growth compared to the stationary phase strongly up-regulated *glu81A, glu16A, glu16B,* and *glu16F* (Fig. 3F). Similarly, the major *C. japonicus* chitinolytic machinery has been characterized via a combination of mutational, biochemical, and secretome studies (48, 49, 61). During exponential growth on necromass compared to glucose, *chi18D, csn46F,* and *chi19A* were strongly up-regulated (Fig. 3E). Previous physiological studies identified Chi18D as an essential chitinase to the degradation of α-chitin and crab shells, followed by Chi18A-C (48). However, mutational analyses of *chi19A* and *csn46F* grown on α-chitin and crab shell resulted in subtle changes in growth compared to wild type (49). Finally, the *C. japonicus* chitinolytic suite also contains LPMO10A, which is known to be physiologically important for *C. japonicus* growth on α-chitin and present in high quantities in the secretome of α-chitin grown cells (61, 62). Interestingly, the expression of the corresponding gene was only up-regulated in the NecLo EXP vs Glc EXP and NecLo STA vs Glc STA comparisons, and not at all up-regulated in NecLo EXP vs NecLo STA. Instead, the gene encoding the cellulose-binding LPMO (*lpmo10B*) was more frequently up-regulated (Tables S11 to S16).

*C. japonicus* was the only species that did not elicit distinguishing differences in the expression of nitrogen utilization-encoding genes based on necromass melanization (Table S23). Assessment of its metabolic activity on various nitrogen sources indicate *C. japonicus* has extremely poor activity on peptides and amino acids, which verifies previous growth studies (32). Rather than necromass-induced changes to the expression of nitrogen utilization-encoding genes, *C. japonicus* exhibited minor differences between exponential growth and the stationary phase on *H. bicolor*. The stationary phase on either necromass type elicited upregulation of *glnA, gltD,* and *gltB*, which encode a glutamine synthetase and two glutamate synthases, respectively. Notably, up-regulation of a glutamine synthetase-encoding gene during the stationary phase is a well-documented occurrence in bacteria and does not appear to be indicative of a unique response to fungal necromass (63–66).

### *S. marcescens* could fill the ecological role of a late-stage necromass degrader

*S. marcescens* exhibited the greatest metabolic activity on the fungal cell wall derivatives and amino acids; however, its growth on necromass was poor (Fig. 1 and 4). The lack of any CAZymes belonging to the families GH5, GH16, GH17, and GH30 is likely the cause for the poor growth since these are typically active on β-1,3- and β-1,6-glucans (25, 28). A previous growth assessment of *S. marcescens* on *Aspergillus nidulans* showed the strain had similarly poor growth dynamics compared to α-chitin and glucose (44). Since the structure of fungal cell walls generally have β-glucans as the outer most layer, the observed poor growth could be due to the limited access to chitin (13). Therefore, we suspect that *S. marcescens* would most likely be a late-stage degrader of necromass after the other microbial decomposers have removed the impeding β-glucans (Fig. 5).

CAZyme-encoding gene expression of *S. marcescens* during exponential growth on glucose compared to the stationary phase was much more modest than that observed for *C. pinensis* (Fig. 2). Only nine CAZyme-encoding genes were up-regulated and mostly included those relevant to chitin and glucan hydrolysis. Considering that *S. marcescens* does not have any CAZymes belonging to the fungal glucan-degrading GH families, the three up-regulated glucanase-encoding genes (which encode 6-phospho-β-glucosidases) are unlikely to be important to fungal necromass decomposition. However, there were up-regulated GH18 class chitinase-encoding genes as well as one *N*-acetyl-β-hexosaminidase encoding gene also up-regulated. Despite a limited and overall growth rate-dependent CAZyme-encoding gene expression response, *S. marcescens* clearly prioritizes the utilization of chitin by rapidly expressing nearly all its chitinase-encoding genes based solely on the growth rate. This preparation for chitin exposure suggests that *S. marcescens* abundance might increase during late-stage decomposition when chitin is potentially the primary remaining nitrogen source remaining. *S. marcescens* has a well-studied chitinase system which includes three chitinases belonging to GH18 and an LPMO from AA10 (67–69), and as possessing a model chitinolytic system (70), *S. marcescens* likely out-competes other microbes during late-stage necromass decay.

Similar to *C. pinensis, S. marcescens* had altered expression of nitrogen utilization-encoding genes on the high vs low melanin necromass. During exponential growth on the low melanin necromass*, S. marcescens* up-regulated almost three times the nitrogen utilization-encoding genes compared to exponential growth on high melanin necromass (Table S23). Since *S. marcescens* growth on necromass was sparse, the expression of several glutamine synthetases may have increased as part of a starvation response (66, 71).

### Concluding remarks

To gain a predictive capacity of necrobiome composition and dynamics, research requires an integrated approach, starting with community characterization and moving toward mechanisms of substrate degradation, followed by experimental studies in community settings. In this study, we have transcriptomically characterized three bacterial species during necromass decomposition and provided a series of hypotheses concerning their contribution to necromass decay *in vivo*. Our results indicate that melanin content of *H. bicolor* necromass elicits the same CAZyme-encoding gene expression responses in *C. japonicus*, *C. pinensis*, and *S. marcescens*. Considering the growth capabilities and gene expression responses collectively, we present a model of different niches for each species during decomposition from which more detailed genetic and biochemical studies of microbial necromass degraders can be built.

## Data Availability

All RNAseq data have been deposited in NCBI GEO (BioProjects GSE149593 and GSE268149). The RNAseq data for *C. japonicus* grown on glucose was obtained from NCBI SRA: SRX8207642, SRX8207643, SRX8207644, SRX8207645, SRX8207646, and SRX8207647. Glucose RNAseq data for *C. pinensis* and *S. marcescens* have been deposited in NCBI SRA (*C. pinensis* SRX24655130, SRX24655128, SRX24655127, SRX24655125, SRX24655124, and SRX24655122; *S. marcescens* SRX24655148, SRX24655147, SRX24655146, SRX24655145, SRX24655144, and SRX24655143). High melanin *H. bicolor* RNAseq data have been deposited in NCBI SRA (*C. japonicus* SRX24655114, SRX24655113, SRX24655112, SRX24655111, SRX24655109, and SRX24655107; *C. pinensis* SRX24655136, SRX24655135, SRX24655134, SRX24655133, SRX24655132, and SRX24655131; *S. marcescens* SRX24655154, SRX24655153, SRX24655152, SRX24655151, SRX24655150, and SRX24655149). Low melanin *H. bicolor* RNAseq data have been deposited in NCBI SRA (*C. japonicus* SRX24655120, SRX24655119, SRX24655118, SRX24655117, SRX24655116, and SRX24655115; *C. pinensis* SRX24655142, SRX24655141, SRX24655140, SRX24655139, SRX24655138, and SRX24655137; *S. marcescens* SRX24655160, SRX24655159, SRX24655158, SRX24655157, SRX24655156, and SRX24655155).

## SUPPLEMENTAL MATERIAL

Supplemental figures and tables aem.01062-24-s0001.pdf Figures S1 and S2; Tables S1, S2, and S23-S27.

Supplemental tables aem.01062-24-s0002.xlsx Tables S3 to S22.

ASM does not own the copyrights to Supplemental Material that may be linked to, or accessed through, an article. The authors have granted ASM a non-exclusive, world-wide license to publish the Supplemental Material files. Please contact the corresponding author directly for reuse.
