# Supplementary material for: Transcriptomic analyses of bacterial growth on fungal necromass reveal different microbial community niches during degradation
Source: Appl Environ Microbiol. 2024 Sep 12;90(10):e01062-24. doi: 10.1128/aem.01062-24 (PMC11497827; doi:10.1128/aem.01062-24)
Supplement: Supplemental figures and tables — Figures S1 and S2; Tables S1, S2, and S23-S27. [file aem.01062-24-s0001.pdf]

Transcriptomic analyses of bacterial growth on fungal necromass reveals different microbial community niches during degradation

Jessica K. Novak[1], Peter G. Kennedy[2], and Jeffrey G. Gardner[1#]

Running Title

Transcriptomic analysis of bacteria grown on fungal necromass

Keywords

Carbohydrate active enzyme, *Cellvibrio japonicus*, *Chitinophaga pinensis*, *Hyaloscypha bicolor*, necromass, *Serratia marcescens*

Author Affiliations

[1]Department of Biological Sciences, University of Maryland - Baltimore County Baltimore, Maryland, USA

[2]Department of Plant and Microbial Biology, University of Minnesota, Minneapolis, Minnesota, USA

Figure S1. Growth analyses of *C. japonicus, C. pinensis*, and *S. marcescens* when provided MOPS minimal media supplemented with 0.2% glucose as the sole carbon source. All growth experiments were completed in biological triplicate, with error bars representing standard deviations, although some are too small to be observed. Optical density measurements were taken using an EPOCH microplate reader.

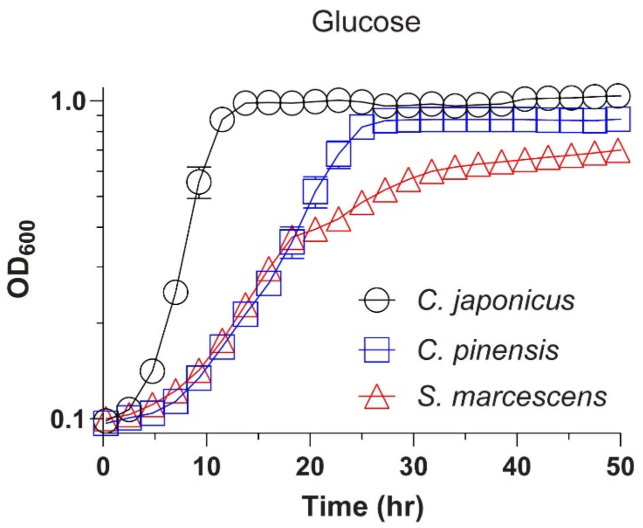

Table S1. Growth dynamics of *C. japonicus, C. pinensis,* and *S. marcescens* on MOPS defined media supplemented with 0.2% (w/v) glucose.

Strain	Lag phase (hours)	Timepoints (T_i and T_f)	Growth rate (gen/hr)	Max OD_{600}
C. japonicus	4	5, 10	0.33 ± 0.03	1.04 ± 0.04
C. pinensis	8.25	12, 23	0.13 ± 0.01	0.88 ± 0.01
S. marcescens	7.5	10, 18	0.12 ± 0.01	0.71 ± 0.06

Table S2. Growth dynamics of *C. japonicus, C. pinensis,* and *S. marcescens* on MOPS defined media supplemented with 1% (w/v) high or low melanin fungal necromass.

Substrate	Strain	Lag phase (hours)	Timepoints (T_i and T_f)	Growth rate (gen/hr)	Max OD_{600}
H. bicolor (low melanin)	*C. japonicus*	11.25	12.5, 26.25	0.17 ± 0.02	0.92 ± 0.05
	C. pinensis	< 10	10, 20.5	0.16 ± 0.01	1.77 ± 0.06
	S. marcescens	29.5	29.5, 43	0.08 ± 0.03	0.12 ± 0.03

H. bicolor (high melanin)	*C. japonicus*	27	27, 29.75	0.37 ± 0.04	1.21 ± 0.1
	C. pinensis	4.5	4.5, 45	0.08 ± 0.01	1.38 ± 0.11
	S. marcescens	31	19, 25.5	0.12 ± 0.03	0.06 ± 0.02

Figure S2. Changes to CAZyme-encoding gene expression during various growth phases on high melanin *H. bicolor*. (**A**) Volcano plot representation of gene expression data for *C. pinensis* during exponential growth on high melanin *H. bicolor* compared to glucose. (**B**) Volcano plot representation of gene expression data for *C. pinensis* grown on high melanin *H. bicolor* during exponential growth compared to stationary phase. (**C**) Volcano plot representation of gene expression data for *C. pinensis* during stationary phase on high melanin *H. bicolor* compared to glucose. (**D**) Heat map showing changes in differential CAZyme gene expression in *C. pinensis*. (**E**) Volcano plot representation of gene expression data for *C. japonicus* during exponential growth on high melanin *H. bicolor* compared to glucose. (**F**) Volcano plot representation of gene expression data for *C. japonicus* grown on high melanin *H. bicolor* during exponential growth compared to stationary phase. (**G**) Volcano plot representation of gene expression data for *C. japonicus* during stationary phase on high melanin *H. bicolor* compared to glucose. (**H**) Heat map showing changes in differential CAZyme gene expression in *C. japonicus*. (**I**) Volcano plot representation of gene expression data for *S. marcescens* during exponential growth on high melanin *H. bicolor* compared to glucose. (**J**) Volcano plot representation of gene expression data for *S. marcescens* grown on high melanin *H. bicolor* during exponential growth compared to stationary phase. (**K**) Volcano plot representation of gene expression data for *S. marcescens* during stationary phase on high melanin *H. bicolor* compared to glucose. (**L**) Heat map showing changes in differential CAZyme gene expression in *S. marcescens*. Open grey circles in the volcano plots represent a single gene and colored symbols represent a CAZyme-encoding gene that correlates to the symbol/color provided in the legend.

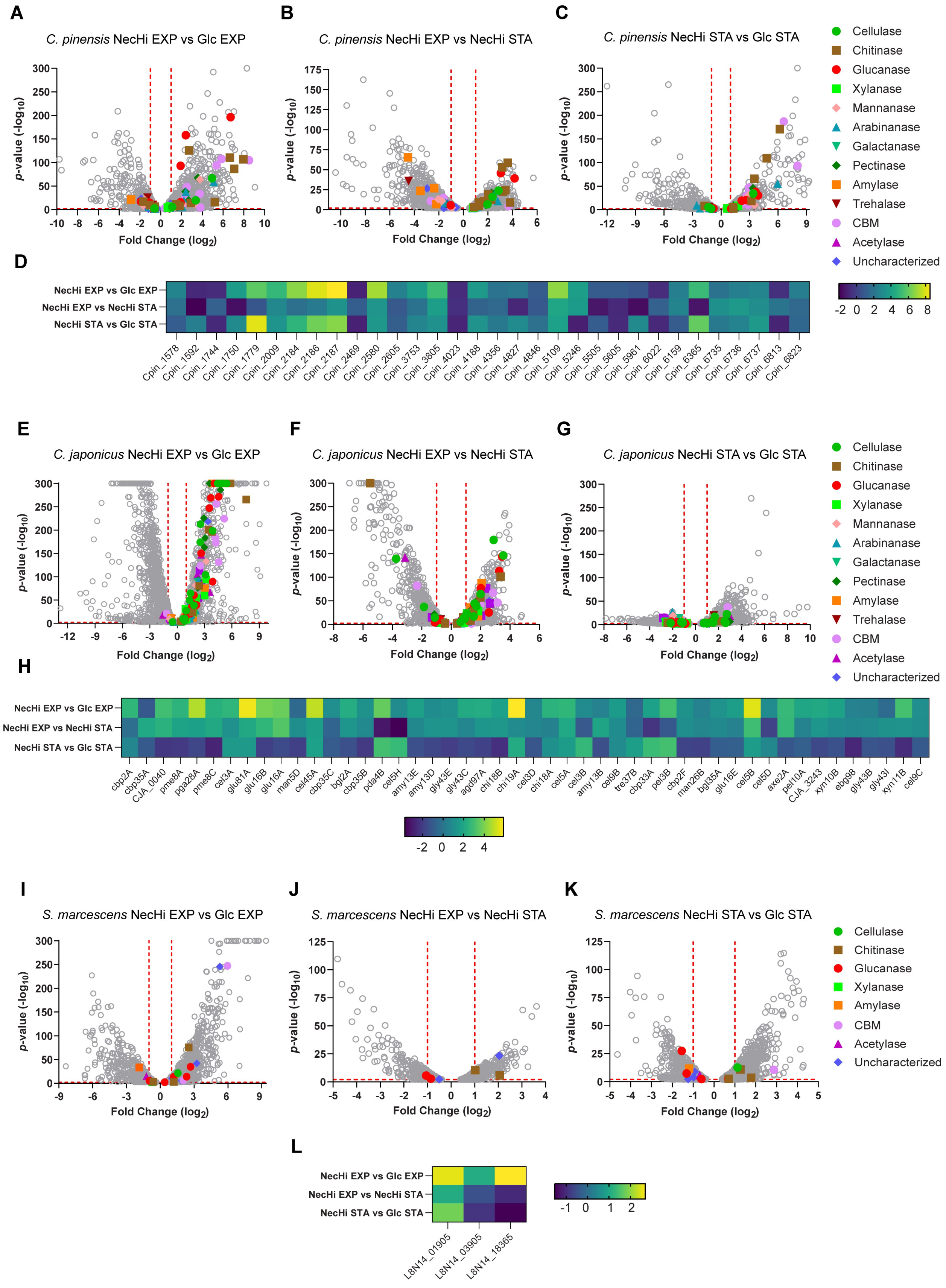
A
C. pinensis NecHi EXP vs Glc EXP
B
C. pinensis NecHi EXP vs NecHi STA
C
C. pinensis NecHi STA vs Glc STA
p-value (-log10)
Fold Change (log2)
Cellulase
Chitinase
Glucanase
Xylanase
Mannanase
Arabinanase
Galactanase
Pectinase
Amylase
Trehalase
CBM
Acetylase
Uncharacterized
D
NecHi EXP vs Glc EXP
NecHi EXP vs NecHi STA
NecHi STA vs Glc STA
Cpin_1578
Cpin_1592
Cpin_1744
Cpin_1750
Cpin_1779
Cpin_2009
Cpin_2184
Cpin_2186
Cpin_2187
Cpin_2469
Cpin_2580
Cpin_2605
Cpin_3753
Cpin_3805
Cpin_4023
Cpin_4188
Cpin_4356
Cpin_4827
Cpin_4846
Cpin_5109
Cpin_5246
Cpin_5505
Cpin_5605
Cpin_5961
Cpin_6022
Cpin_6159
Cpin_6365
Cpin_6735
Cpin_6736
Cpin_6737
Cpin_6813
Cpin_6823
-2 0 2 4 6 8
E
C. japonicus NecHi EXP vs Glc EXP
F
C. japonicus NecHi EXP vs NecHi STA
G
C. japonicus NecHi STA vs Glc STA
p-value (-log10)
Fold Change (log2)
Cellulase
Chitinase
Glucanase
Xylanase
Mannanase
Arabinanase
Galactanase
Pectinase
Amylase
Trehalase
CBM
Acetylase
Uncharacterized
H
NecHi EXP vs Glc EXP
NecHi EXP vs NecHi STA
NecHi STA vs Glc STA
cbp2A
cbp35A
CJA_0040
pme8A
pga28A
pme8C
cel3A
glu81A
glu16B
glu16A
man5D
cel45A
cbp35C
bgl2A
cbp35B
pda4B
cel5H
amy13E
amy13D
gly43E
gly43C
agd97A
chi18B
chi19A
cel3D
chi18A
cel5A
cel3B
amy13B
cel6B
tre37B
cbp33A
pel3B
cbp2F
man26B
bgl35A
glu16E
cel5B
cel5D
axe2A
pel10A
CJA_3243
xyn10B
ebg98
gly43B
gly43I
xyn11B
cel9C
-2 0 2 4
I
S. marcescens NecHi EXP vs Glc EXP
J
S. marcescens NecHi EXP vs NecHi STA
K
S. marcescens NecHi STA vs Glc STA
p-value (-log10)
Fold Change (log2)
Cellulase
Chitinase
Glucanase
Xylanase
Amylase
CBM
Acetylase
Uncharacterized
L
NecHi EXP vs Glc EXP
NecHi EXP vs NecHi STA
NecHi STA vs Glc STA
L8N14_01905
L8N14_03905
L8N14_18365
-1 0 1 2

Table S23. Exponential growth on glucose compared to stationary phase elicits up regulation of different nitrogen utilization-encoding genes than exponential growth on necromass compared to stationary phase. All genes listed were up regulated under the specified conditions and have a *p*-value $\leq$ 0.01.

	Glc EXP vs Glc STA	NecLo EXP vs Glc EXP	NecHi EXP vs Glc EXP	NecLo STA vs Glc STA	NecHi STA vs Glc STA
C. pinensis	*cpin_7211* (glutamate ammonia-ligase)	--	--	--	--
	cpin_1662 (glutamate synthetase)	--	--	--	--
	cpin_1924 (threonine dehydratase)	--	--	--	--
	cpin_0731 (glutamate synthase)	--	--	--	--
	cpin_1853 (histidine ammonia-lyase)	--	--	--	--
	cpin_0730 (glutamate synthase)	--	--	--	--
	--	*cpin_2006* (histidine ammonia-lyase)	--	--	--
	--	*cpin_3374* (alanine de-hydrogenase)	--	--	--
	--	*cpin_1695* (nitropropane dioxygenase)	*cpin_1695* (nitropropane dioxygenase)	*cpin_1695* (nitropropane dioxygenase)	--
	--	*cpin_0230* (nitrogen-fixing domain)	--	*cpin_0230* (nitrogen-fixing domain)	*cpin_0230* (nitrogen-fixing domain)
		--		*cpin_4284* (nitro-reductase)	--

	--<br><br>--	--	--<br><br>--	*cpin_5012* (nitro- reductase)	--
C. japonicus	*gdhA* (glutamate de- hydrogenase)	--	--	--	--
	ilvA (threonine ammonia- lyase)	--	--	--	--
	adgA (glutamine- dependent NAD+ synthetase)	--	--	--	--
	cja_1559 (nitro-- reductase)	--	--	--	--
	--	*cja_2161* (NAD-- dependent glutamate de- hydrogenase)	*cja_2161* (NAD-- dependent glutamate de- hydrogenase)	--	--
	--	*ald* (alanine de- hydrogenase)	*ald* (alanine de- hydrogenase)	--	--
	--	*cja_3392* (nitrogen regulatory protein P-II)	*cja_3392* (nitrogen regulatory protein P-II)	*cja_3392* (nitrogen regulatory protein P-II)	*cja_3392* (nitrogen regulatory protein P-II)
	--	*ntrC* (nitrogen regulatory protein NR(I))	*ntrC* (nitrogen regulatory protein NR(I))	*ntrC* (nitrogen regulation protein NR(I))	*ntrC* (nitrogen regulation protein NR(I))
	--	*norB* (nitric oxide reductase)	*norB* (nitric oxide reductase)	--	--
	--	*cja_3390* (nitrogen regulatory protein P-II)	*cja_3390* (nitrogen regulatory protein P-II)	*cja_3390* (nitrogen regulatory protein P-II)	*cja_3390* (nitrogen regulatory protein P-II)
	--	*nirB*	*nirB*	*nirB* (nitrite reductase)	*nirB* (nitrite reductase)

		(nitrite reductase)	(nitrite reductase)		
	--	*nirD* (nitrite reductase)	*nirD* (nitrite reductase)	*nirD* (nitrite reductase)	*nirD* (nitrite reductase)
	glnA (glutamine synthetase)	--	--	*glnA* (glutamine synthetase)	*glnA* (glutamine synthetase)
	--	--	--	*cja_3536* (NtrC two component system)	--
	gltD (glutamate synthase)	--	--	*gltD* (glutamate synthase)	*gltD* (glutamate synthase)
	--	--	--	*gltB* (glutamate synthase)	*gltB* (glutamate synthase)
	--	--	--	--	*cja_1973* (glutamate synthase)
S. marcescens	*L8N14_00130* (glutamine amido-transferase)	--	--	--	--
	L8N14_17335 (aspartate ammonia-lyase)	--	--	--	--
	L8N14_21270 (threonine ammonia-lyase)	--	--	--	--
	--	*L8N14_11925* (nitrogen assimilation transcriptional regulator)	*L8N14_11925* (nitrogen assimilation transcriptional regulator)	--	--
	--	*L8N14_06450* (glutamine synthetase)	*L8N14_06450* (glutamine synthetase)	--	--
	--	*L8N14_10750* (nitrate reductase)	--	--	--
		L8N14_04185	*L8N14_04185*		*L8N14_04185*

--	(L-serine ammonia-lyase)	(L-serine ammonia-lyase)	--	(L-serine ammonia-lyase)
--	*L8N14_21470* (glutamate de-hydrogenase)	*L8N14_21470* (glutamate de-hydrogenase)	--	--
--	*L8N14_16415* (glutamine synthetase)	--	--	--
--	*L8N14_18380* (glutamine-hydrolyzing GMP synthase)	--	--	--
--	*L8N14_13885* (ethanolamine ammonia-lyase)	--	--	--
--	*L8N14_10745* (nitrate reductase)	--	--	--
--	*L8N14_18910* (nitrite/nitrate two-component sensor)	--	*L8N14_18910* (nitrite/nitrate two-component sensor)	--
--	*L8N14_15185* (glutamate synthase)	--	--	--
L8N14_21965 (nitrite reductase)	--	--	*L8N14_21965* (nitrite reductase)	*L8N14_21965* (nitrite reductase)
L8N14_05810 (carbon-nitrogen hydrolase)	--	--	*L8N14_05810* (carbon-nitrogen hydrolase)	*L8N14_05810* (carbon-nitrogen hydrolase)
--	--	--	*L8N14_24000* (threonine/ serine dehydratase)	--
L8N14_23630 (nitrogen regulation protein NR(I))	--	--	*L8N14_23630* (nitrogen regulation protein NR(I))	*L8N14_23630* (nitrogen regulation protein NR(I))

	L8N14_12535 (glutamine synthetase)	--	--	*L8N14_12535* (glutamine synthetase)	*L8N14_12535* (glutamine synthetase)
	--	--	--	*L8N14_03515* (glutamate synthase)	--

Table S24. Phenotypic microarray assay of *C. japonicus, C. pinensis,* and *S. marcescens* metabolic activity on various carbon sources tested on PM1. Table corresponds to Figure 4 panels A-C. Metabolic activity was determined using dye reduction kinetic curves from an OmniLog where activity is positively correlated with the numeric value.

**A1** Negative Control	**A2** L-Arabinose	**A3** *N*-Acetyl-D-Gluco-samine	**A4** D-Saccharic Acid	**A5** Succinic Acid	**A6** D-Galactose	**A7** L-Aspartic Acid	**A8** L-Proline	**A9** D-Alanine	**A10** D-Trehalose	**A11** D-Mannose	**A12** Dulcitol
**B1** D-Serine	**B2** D-Sorbitol	**B3** Glycerol	**B4** L-Fucose	**B5** D-Glucuronic Acid	**B6** D-Gluconic Acid	**B7** D,L-α-Glycerol Phosphate	**B8** D-Xylose	**B9** L-Lactic Acid	**B10** Formic Acid	**B11** D-Mannitol	**B12** L-Glutamic Acid
**C1** D-Glucose-6-Phosphate	**C2** D-Galactonic Acid-γ-Lactone	**C3** D,L-Malic Acid	**C4** D-Ribose	**C5** Tween 20	**C6** D-Rhamnose	**C7** D-Fructose	**C8** Acetic Acid	**C9** α-D-Glucose	**C10** Maltose	**C11** D-Melibiose	**C12** Thymidine
**D1** L-Asparagine	**D2** D-Aspartic Acid	**D3** D-Gluco-saminic Acid	**D4** 1,2-Propanediol	**D5** Tween 40	**D6** α-Keto-Glutaric Acid	**D7** α-Keto-Butyric Acid	**D8** α-Methyl-D-Galactoside	**D9** α-D-Lactose	**D10** Lactulose	**D11** Sucrose	**D12** Uridine
**E1** L-Glutamine	**E2** m-Tartaric Acid	**E3** D-Glucose-1-Phosphate	**E4** D-Fructose-6-Phosphate	**E5** Tween 80	**E6** α-Hydroxy Glutaric Acid-γ-Lactone	**E7** α-Hydroxy Butyric Acid	**E8** β-Methyl-D-Glucoside	**E9** Adonitol	**E10** Maltotriose	**E11** 2-Deoxy Adenosine	**E12** Adenosine
**F1** Glycyl-L-Aspartic Acid	**F2** Citric Acid	**F3** Myo-Inositol	**F4** D-Threonine	**F5** Fumaric Acid	**F6** Bromo-Succinic Acid	**F7** Propionic Acid	**F8** Mucic Acid	**F9** Glycolic Acid	**F10** Glyoxylic Acid	**F11** D-Cellobiose	**F12** Inosine
**G1** Glycyl-L-Glutamic Acid	**G2** Tri-carbballylic Acid	**G3** L-Serine	**G4** L-Threonine	**G5** L-Alanine	**G6** L-Alanyl Glycine	**G7** Acetoacetic Acid	**G8** *N*-Acetyl-β-D-Manno-samine	**G9** Mono Methyl Succinate	**G10** Methyl Pyruvate	**G11** D-Malic Acid	**G12** L-Malic Acic
**H1** Glycyl-L-Proline	**H2** p-Hydroxy Phenyl Acetic Acid	**H3** m-Hydroxy Phenyl Acetic Acid	**H4** Tyramine	**H5** D-Psicose	**H6** L-Lyxose	**H7** Glucuron-amide	**H8** Pyruvic Acid	**H9** L-Galactonic Acid-γ-Lactone	**H10** D-Galactur-onic Acid	**H11** Phenylethyl-amine	**H12** 2-Amino-ethanol

Table S25. Phenotypic microarray assay of *C. japonicus, C. pinensis,* and *S. marcescens* metabolic activity on various carbon sources tested on PM2. Table corresponds to Figure 4 panels D-F. Metabolic activity was determined using dye reduction kinetic curves from an OmniLog where activity is positively correlated with the numeric value.

**A1**<br>Negative Control	**A2**<br>Chondroitin Sulfate C	**A3**<br>α-Cyclo-dextrin	**A4**<br>β-Cyclo-dextrin	**A5**<br>γ-Cyclo-dextrin	**A6**<br>Dextrin	**A7**<br>Gelatin	**A8**<br>Glycogen	**A9**<br>Inulin	**A10**<br>Laminarin	**A11**<br>α-Mannan	**A12**<br>Pectin
**B1**<br>*N*-Acetyl-Galacto-samine	**B2**<br>*N*-Acetyl Neura-minic Acid	**B3**<br>β-D-Allose	**B4**<br>Amygdalin	**B5**<br>D-Arabinose	**B6**<br>D-Arabitol	**B7**<br>L-Arabitol	**B8**<br>Arbutin	**B9**<br>2-Deoxy-D-Ribose	**B10**<br>i-Erythritol	**B11**<br>D-Fucose	**B12**<br>3-0-β-D-Galacto-pyranosyl-D-Arabinose
**C1**<br>Gentiobiose	**C2**<br>L-Glucose	**C3**<br>Lactitol	**C4**<br>D-Melezitose	**C5**<br>Maltitol	**C6**<br>α-Methyl-D-Glucoside	**C7**<br>β-Methyl-D-Galactoside	**C8**<br>3-Methyl Glucose	**C9**<br>β-Methyl-D-Glucuronic Acid	**C10**<br>α-Methyl-D-Mannoside	**C11**<br>β-Methyl-D-Xyloside	**C12**<br>Palatinose
**D1**<br>D-Raffinose	**D2**<br>Salicin	**D3**<br>Sedo-heptulosan	**D4**<br>L-Sorbose	**D5**<br>Stachyose	**D6**<br>D-Tagatose	**D7**<br>Turanose	**D8**<br>Xylitol	**D9**<br>*N*-Actyl-D-Gluco-saminitol	**D10**<br>γ-Amino-Butyric Acid	**D11**<br>δ-Amino-Valeric Acid	**D12**<br>Butyric Acid
**E1**<br>Capric Acid	**E2**<br>Caproic Acid	**E3**<br>Citraconic Acid	**E4**<br>Citramalic Acid	**E5**<br>D-Glucosamine	**E6**<br>2-Hydroxy Benzoic Acid	**E7**<br>4-Hydroxy Benzoic Acid	**E8**<br>β-Hydroxy Butyric Acid	**E9**<br>Glycolic Acid	**E10**<br>α-Keto-Valeric Acid	**E11**<br>Itaconic Acid	**E12**<br>5-Keto-D-Gluconic Acid
**F1**<br>D-Lactic Acid Methyl Ester	**F2**<br>Malonic Acid	**F3**<br>Melibionic acid	**F4**<br>Oxalic Acid	**F5**<br>Oxalomalic Acid	**F6**<br>Quinic Acid	**F7**<br>D-Ribono-1,4-Lactone	**F8**<br>Sebacic Acid	**F9**<br>Sorbic Acid	**F10**<br>Succinamic Acid	**F11**<br>D-Tartaric Acid	**F12**<br>L-Tartaric Acid
**G1**<br>Acetamide	**G2**<br>L-Alaninamide	**G3**<br>*N*-Acetyl-L-Glutamic Acid	**G4**<br>L-Arginine	**G5**<br>Glycine	**G6**<br>L-Histidine	**G7**<br>L-Homoserine	**G8**<br>Hydroxy-L-Proline	**G9**<br>L-Isoleucine	**G10**<br>L-Leucine	**G11**<br>L-Lysine	**G12**<br>L-Methionine
**H1**<br>L-Ornithine	**H2**<br>L-Phenyl-alanine	**H3**<br>L-Pyro-glutamic Acid	**H4**<br>L-Valine	**H5**<br>D,L-Carnitine	**H6**<br>Sec-Butylamine	**H7**<br>D,L-Octopamine	**H8**<br>Putrescine	**H9**<br>Dihydroxy Acetone	**H10**<br>2,3-Butanediol	**H11**<br>2,3-Butanedione	**H12**<br>3-Hydroxy-2-Butanone

Table S26. Phenotypic microarray assay of *C. japonicus, C. pinensis,* and *S. marcescens* metabolic activity on various nitrogen sources tested on PM3. Table corresponds to Figure 4 panels G-I. Metabolic activity was determined using dye reduction kinetic curves from an OmniLog where activity is positively correlated with the numeric value.

**A1**<br>Negative Control	**A2**<br>Ammonia	**A3**<br>Nitrite	**A4**<br>Nitrate	**A5**<br>Urea	**A6**<br>Biuret	**A7**<br>L-Alanine	**A8**<br>L-Arginine	**A9**<br>L-Asparagine	**A10**<br>L-Aspartic Acid	**A11**<br>L-Cysteine	**A12**<br>L-Glutamic Acid
**B1**<br>L-Glutamine	**B2**<br>Glycine	**B3**<br>L-Histidine	**B4**<br>L-Isoleucine	**B5**<br>L-Leucine	**B6**<br>L-Lysine	**B7**<br>L-Methionine	**B8**<br>L-Phenylalanine	**B9**<br>L-Proline	**B10**<br>L-Serine	**B11**<br>L-Threonine	**B12**<br>L-Tryptophan
**C1**<br>L-Tyrosine	**C2**<br>L-Valine	**C3**<br>D-Alanine	**C4**<br>D-Asparagine	**C5**<br>D-Aspartic Acid	**C6**<br>D-Glutamic Acid	**C7**<br>D-Lysine	**C8**<br>D-Serine	**C9**<br>D-Valine	**C10**<br>D-Citrulline	**C11**<br>D-Homoserine	**C12**<br>D-Ornithine
**D1**<br>*N*-Acetyl-L-Glutamic Acid	**D2**<br>*N*-Phthaloyl-L-Glutamic Acid	**D3**<br>L-Pyroglutamic Acid	**D4**<br>Hydroxylamine	**D5**<br>Methylamine	**D6**<br>*N*-Amylamine	**D7**<br>*N*-Butylamine	**D8**<br>Ethylamine	**D9**<br>Ethanolamine	**D10**<br>Ethylenediamine	**D11**<br>Putrescine	**D12**<br>Agmatine
**E1**<br>Histamine	**E2**<br>β-Phenylethylamine	**E3**<br>Tyramine	**E4**<br>Acetamide	**E5**<br>Formamide	**E6**<br>Glucuronamide	**E7**<br>D,L-Lactamide	**E8**<br>D-Glucosamine	**E9**<br>D-Galactosamine	**E10**<br>D-Mannosamine	**E11**<br>*N*-Acetyl-D-Glucosamine	**E12**<br>*N*-Acetyl-D-Galactosamine
**F1**<br>*N*-Acetyl-D-Mannosamine	**F2**<br>Adenine	**F3**<br>Adenosine	**F4**<br>Cytidine	**F5**<br>Cytosine	**F6**<br>Guanine	**F7**<br>Guanosine	**F8**<br>Thymine	**F9**<br>Thymidine	**F10**<br>Uracil	**F11**<br>Uridine	**F12**<br>Inosine
**G1**<br>Xanthine	**G2**<br>Xanthosine	**G3**<br>Uric Acid	**G4**<br>Alloxan	**G5**<br>Allantoin	**G6**<br>Parabanic Acid	**G7**<br>D,L-α-Amino-*N*-Butyric Acid	**G8**<br>γ-Amino-*N*-Butyric Acid	**G9**<br>ε-Amino-*N*-Caproic Acid	**G10**<br>D,L-α-Amino-Caprylic Acid	**G11**<br>δ-Amino-*N*-Valeric Acid	**G12**<br>α-Amino-*N*-Valeric Acid
**H1**<br>Ala-Asp	**H2**<br>Ala-Gln	**H3**<br>Ala-Glu	**H4**<br>Ala-Gly	**H5**<br>Ala-His	**H6**<br>Ala-Leu	**H7**<br>Ala-Thr	**H8**<br>Gly-Asn	**H9**<br>Gly-Gln	**H10**<br>Gly-Glu	**H11**<br>Gly-Met	**H12**<br>Met-Ala

Table S27. Thermochemolysis-GCMS of intermediately melanized *Hyaloscypha bicolor* demonstrates the initial necromass is largely comprised of lipids, carbohydrates, and unspecified compounds.

Compound	Average Relative Abundance
Aromatic	0.0048 ± 0.0002
Lipid	0.3615 ± 0.0161
Nitrogen-containing	0.1267 ± 0.108
Sterol	0.0014 ± 0.0003
Carbohydrates	0.2915 ± 0.0124
Unspecified	0.2140 ± 0.0163
